# Synergy Between Photon‐to‐Phonon Pathway and Active Lattice Oxygen Enables Efficient and Stable Syngas Synthesis

**DOI:** 10.1002/anie.9236832

**Published:** 2026-08-05

**Authors:** Chengzhi Guo, Apoorv Jain, Junrun Feng, Xinyu Li, Xinru Li, Shuya Jia, Juncong Wang, Leirun Chen, Xinjie Luo, Xiaolei Zhang, Xiyi Li, Yang Lan

**Affiliations:** ^1^ Department of Chemical Engineering University College London London UK; ^2^ Department of Chemical and Process Engineering University of Strathclyde Glasgow UK; ^3^ School of Science School of Chip Industry Hubei University of Technology Wuhan Hubei China; ^4^ College of New Energy Xi'an Shiyou University Xi'an Shaanxi China

**Keywords:** active lattice oxygen, dry reforming of methane, photon‐to‐phonon conversion, photothermo catalysis

## Abstract

Light‐driven dry reforming of methane (DRM) offers a promising route for syngas synthesis while simultaneously mitigating greenhouse gas emissions of CO_2_ and CH_4_. However, the attractive mild‐temperature operating window imposes kinetic constraints on C─H/C═O activation and promotes thermodynamic tendencies for coke formation, resulting in limited efficiency and stability. Herein, manganese oxide (MnO_x_) is employed as a multifunctional support to integrate the classic Rh catalytic center, establishing a new benchmark photothermo catalyst for DRM. The system achieves record‐high syngas production rates (H_2_: 948 mmol g^−1^ h^−1^; CO: 992 mmol g^−1^ h^−1^) without external heating, alongside exceptional long‐term stability (∼500 h). These production rates and stability also surpass conventional thermocatalysts in similar temperature ranges, with stability exceeding most thermocatalysts by an order of magnitude. Under a separate low‐conversion, high‐gas hourly space velocity (GHSV) protocol, a light‐to‐chemical efficiency (29.5%) can also be reached. MnO_x_ functions as a broadband light harvester, generating a localized thermal field at the micrometre‐scale via an efficient photon‐to‐phonon pathway to facilitate C─H bond activation on Rh. Concurrently, its active lattice oxygen enables a dynamic O_L_‐O_V_ cycle for timely removal of C* intermediates and C═O activation. This work underscores the critical role of support engineering in advancing light‐driven DRM.

## Graphical abstract

This work reports the design of a multifunctional MnO_x_ support coupled with classic Rh catalytic centers for light‐driven dry reforming of methane. Benefiting from efficient photon‐to‐phonon conversion pathway and a dynamic O_L_‐O_V_ cycle, the catalyst achieves high syngas production rates and light‐to‐chemical energy efficiency without external heating, as well as exceptional long‐term stability.

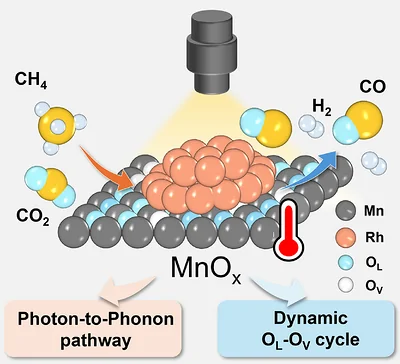

## 1 Introduction

Dry reforming of methane (DRM) provides a promising pathway for valuable syngas synthesis (CH_4_ + CO_2_ → 2CO + 2H_2_, Δ*H* = + 247 kJ mol^−1^), offering significant potential for industrial feedstock production while addressing greenhouse concerns [1, 2, 3]. However, the inert nature of CH_4_ and CO_2_ (C─H bond energy: 434 kJ mol^−1^, C═O bond energy: 750 kJ mol^−1^) typically requires high temperatures (>800°C) to overcome the kinetic barriers in thermocatalysis, resulting in substantial energy consumption with a moderate catalyst lifetime and a positive net carbon footprint [1, 2, 4, 5, 6].

Light‐driven DRM has recently emerged as a promising alternative, not only due to its ultimate goal of utilizing abundant and clean solar energy as the sole driving force, but also because it enables milder reaction temperatures through the involvement of photoexcited carriers [3, 7, 8, 9, 10, 11, 12, 13]. However, there remain major challenges in this area: (1) Research has mainly focused on engineering catalytic centres, such as single atomic sites [14], nanocluster/nanoalloy [12], or high‐entropy alloys [9], despite the critical role of supports in facilitating the catalytic cycle through lattice oxygen (O_L_) or oxygen vacancies (O_V_). (2) The reliance on highly energetic carriers for substrate activation, such as those generated by large bandgap semiconductors [3, 9] or plasmonic effect excitations [15, 16, 17], restricts utilisation to a narrow portion of the solar spectrum, primarily in the UV and/or visible regions. Consequently, these two challenges result in the low overall production rates of H_2_ and CO (many catalysts even < 0.1 mol g^−1^ h^−1^) [3, 13, 14, 18, 19, 20, 21, 22], and very modest light‐to‐chemical efficiency (typically 1%‐10%) [9, 11, 13, 14, 23]. (3) From a thermodynamic perspective, operating DRM at lower temperatures introduces an inherent tendency to coke formation via methane decomposition (CH_4_ → C(s) + 2H_2_, Δ*H* = + 75 kJ mol^−1^) and CO disproportionation (2CO → C(s) + CO_2_, Δ*H* = −171 kJ mol^−1^) [2, 24, 25]. As a result, coke accumulation remains a critical issue for catalyst deactivation, leading most systems to operate for only short durations (e.g., 5–150 h) [3, 8, 9, 11, 12, 18, 19, 20, 21, 22, 23, 26], and gradual declines in productivity can even be observed in some cases [3, 7, 9, 10, 11, 14].

Our work emphasises support manipulation to overcome current limitations in light‐driven DRM. First, a photon‐to‐phonon conversion pathway has been designed to transform often‐neglected photons (e.g., IR photons) into effective energy carriers, enabling utilization of the full solar spectrum. Second, to enhance stability, active O_L_ is leveraged to promote dynamic redox cycles, facilitating surface carbon species consumption and CO_2_ activation.

Multifunctional manganese oxides (MnO_x_) have emerged as an ideal support platform due to their unique electronic and structural properties. Their multiple oxidation states (Mn^2+^/Mn^3+^/Mn^4+^) not only enable bandgap tunability and broad light absorption (up to 2500 nm) [27, 28, 29, 30, 31, 32, 33], but also enhance the flexibility and reactivity of O_L_ [34]. This high oxygen mobility facilitates the formation of O_V_, which can introduce extra energy levels in the bandgap to enhance light absorption [35, 36, 37]. Moreover, the dynamic exchange between O_L_ and O_v_ plays a crucial role in substrate activation through mechanisms such as the Mars‐van Krevelen pathway [38, 39]. After screening a series of representative transition metals commonly used as catalytic centres in DRM [40, 41, 42, 43, 44, 45], the assembly of Rh and MnO_x_ yields an exceptionally efficient and stable catalyst for light‐driven DRM. The unique Rh/MnO_x_ interface delivers unprecedented H_2_ and CO production rates of nearly 1 mol g^−1^ h^−1^, with a light‐to‐chemical efficiency of 29.5%. More importantly, the catalyst exhibits high stability for 502 h with no detectable graphitic/filamentous coke accumulation under the Raman and TPO‐MS analyses.

## 2 Results and Discussion

### 2.1 Light‐Driven DRM Performance

Transition metals (Ni, Rh, Pt, Pd, Au, and Ag), known for promoting C─H activation in thermocatalytic DRM [40, 41, 42, 43, 44, 45], were incorporated into the MnO_x_ support using a modified citric acid combustion method [46]. Light‐driven DRM reactions were then carried out in a flow reactor at atmospheric pressure without any external heating. As shown in Figure 1a, distinct differences in H_2_ and CO production rates are observed, following the trend: Rh > Pd > Ni > Pt > Au > Ag, likely due to the superior C─H activation capability of Rh [1, 47, 48]. The different performances of the screened catalysts were further investigated (Figure S1 and Note S1). The deviation of the H_2_/CO ratio from unity is likely attributed to the occurrence of the reverse water‐gas shift (RWGS) reaction [1, 9]. Therefore, Rh is selected as the optimized catalytic centre to combine with the MnO_x_ support for subsequent investigations.

**FIGURE 1:**
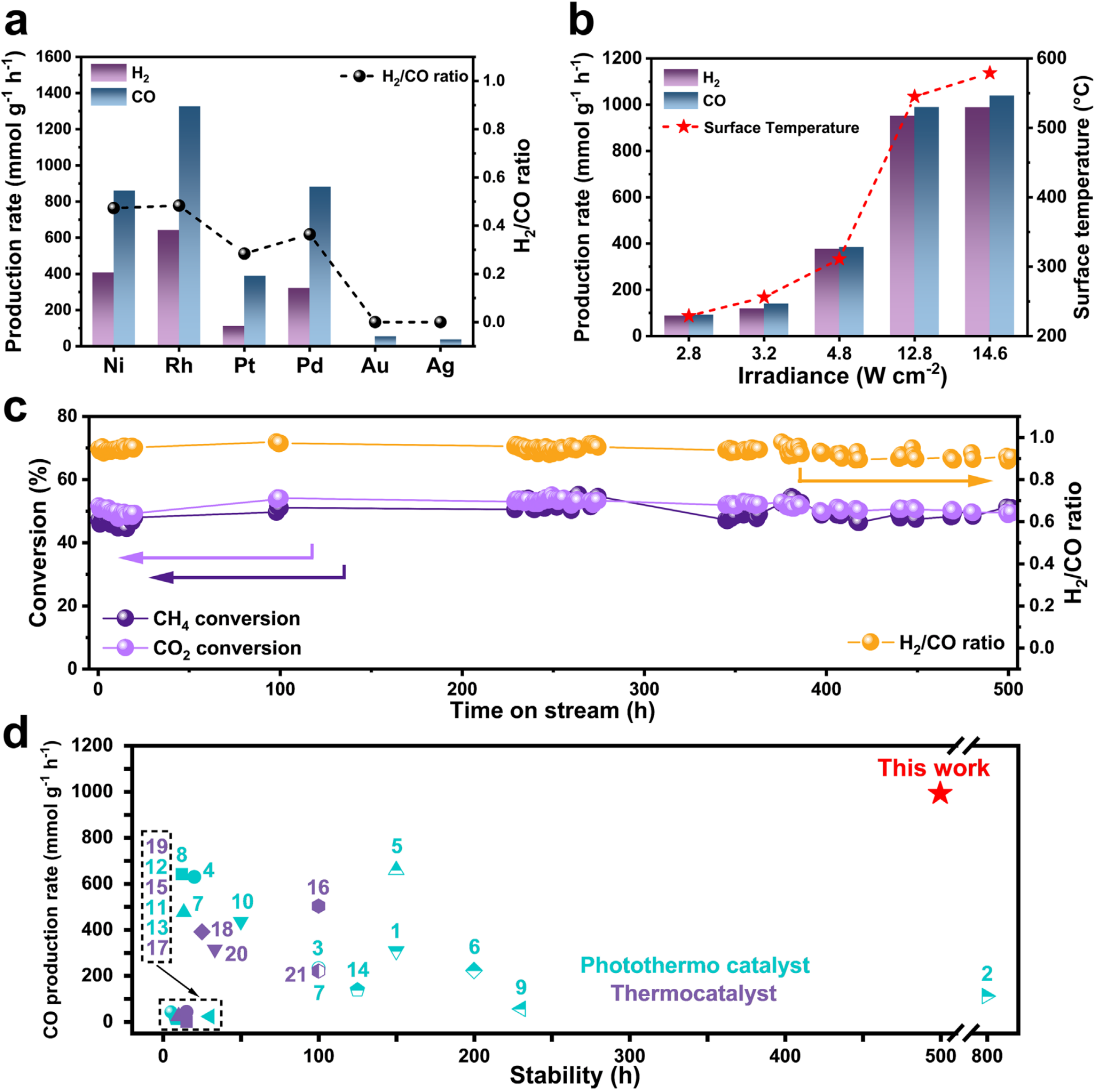
(a) Production rates of H_2_ and CO and H_2_/CO ratio of M_1_/MnO_x_ (M_1_ = Ni, Rh, Pd, Pt, Au, and Ag) taken at 40 min time‐on‐stream. (b) Production rates of H_2_ and CO and surface temperature of Rh_2.5_/MnO_x_ in terms of different irradiances. (c) Long‐term stability test of Rh_2.5_/MnO_x_ under the optimized reaction conditions. (d) Presentation of the Rh_2.5_/MnO_x_ catalyst (red) with other representative photothermo catalysts (cyan) and thermocatalysts (purple) operating under similar irradiance within a comparable temperature range (350–580°C). (The symbols in Figure 1d represent the CO production rates measured at the final run of the stability test. 1: CoNiRuRhPd/SrTiO_3_ [9]; 2: Ni/CeO_2_ [10]; 3: Rh/SrTiO_3_ [11]; 4: NiFe nanoalloy/Al_2_O_3_ [12]; 5: Ru/LaMnFeO [8]; 6: CoNiFeZnCr‐HEO/Rh [7]; 7: Ru/TiO_2_ [49]; 8: RuNi single‐atom alloy [23]; 9: Ni/CeO_2_ [14]; 10: Ni/CeO_2‐x_ [26]; 11: Rh/TiO_2_ [20]; 12: Ni/SiO_2_ [21]; 13: Ni/Ga_2_O_3_ [22]; 14: Ru/BaTiO_3_ [50]; 15: Pt‐CeO_2_ [40];16: Ni/CeO_2_ nanorod [51]; 17: Ni‐Si/ZrO_2_ [41]; 18: Ni‐Fe/Mg(Al)O [52]; 19: Ni@SiO_2_‐ZrO_2_ [53]; 20: Ni/SiO_2_ [54]; 21: NiZnC/Al_2_O_3_ [55]. Detailed reaction conditions can be found in Tables S2 and S3, and a zoomed‐in area (black rectangle) is shown in Figure S15).

The effect of Rh loading amount was investigated, as shown in Figure S2. Notably, MnO_x_ alone shows no catalytic activity, highlighting the essential role of Rh in initiating the DRM reaction, as discussed later. With increasing Rh content, the H_2_ and CO production rates display a volcano‐shaped trend, reaching a maximum at a Rh:Mn molar ratio of 2.5:100. Further Rh loading results in performance decline, probably due to agglomeration of Rh species, which reduces accessible active sites or limits light penetration to the MnO_x_ support via scattering [8, 56, 57, 58]. Therefore, the sample prepared with a Rh:Mn molar ratio of 2.5:100 is selected for subsequent studies (denoted as Rh_2.5_/MnO_x_ unless otherwise specified). It should be noted that the term *MnO_x_* refers to Mn_2_O_3_ before the DRM reaction and MnO after the reaction, owing to the dynamic structural transformation discussed in detail in the later section. The actual Rh loading of this optimized sample, determined by Inductively Coupled Plasma Optical Emission Spectroscopy (ICP‐OES), is 2.68 wt% (Table S1).

The influence of reactant composition on the light‐driven DRM over Rh_2.5_/MnO_x_ was examined, as shown in Figures S3 and S4. The gas hourly space velocity (GHSV) was maintained at 192,000 mL h^−1^ g_cat_^−1^, while the ratio of CH_4_/CO_2_/N_2_/Ar was varied from 1.5/1.5/2/27 to 15/15/2/0. At the beginning, the H_2_ and CO production rates increase proportionally with the rising reactant concentrations, from 430 and 410 mmol g^−1^ h^−1^ at the CH_4_/CO_2_/N_2_/Ar ratio of 1.5/1.5/2/27 to 1187 and 1513 mmol g^−1^ h^−1^ at the CH_4_/CO_2_/N_2_/Ar ratio of 8/8/2/14 (Figure S3). Interestingly, when the ratio further changes to 15/15/2/0, the H_2_ production rate decreases to 1037 mmol g^−1^ h^−1^ while the CO production rate continuously increases to 1810 mmol g^−1^ h^−1^. The H_2_/CO ratio steadily decreases from 1.05 to 0.57 as the ratio of CH_4_/CO_2_/N_2_/Ar changes from 1.5/1.5/2/27 to 15/15/2/0. This trend indicates an increasing contribution from the RWGS reaction at higher CO_2_ concentrations [9]. Moreover, the CH_4_ and CO_2_ conversion gradually decreases from ∼65% to ∼25% as the ratio changes from 1.5/1.5/2/27 to 15/15/2/0 (Figure S4). Based on a balance between catalytic activity, the H_2_/CO ratio, and the CH_4_/CO_2_ conversion, a feed composition of CH_4_/CO_2_/N_2_/Ar = 4/4/2/22 is selected for further performance tests.

Next, the flow rate of Ar was manipulated to control the residence time of reactants (Figures S5 and S6). As the flow rate of Ar increases from 0 to 42 mL min^−1^, both H_2_ and CO production rates exhibit a volcanic trend. They reach a maximum (H_2_: 952 mmol g^−1^ h^−1^; CO: 990 mmol g^−1^ h^−1^) at an Ar flow rate of 22 mL min^−1^, which corresponds to CH_4_ and CO_2_ conversions of 51.8 and 51.9%, attributed to enhanced mass transfer at higher flow rates [24, 59]. However, further increasing the flow rate of Ar from 22 mL min^−1^ to 42 mL min^−1^ greatly dilutes the concentrations of CH_4_ and CO_2_, which decreases their adsorption on the catalyst surface [24]. The lower concentrations lead to a decrease in H_2_/CO production rates as well as CH_4_/CO_2_ conversion. It is worth noting that the H_2_/CO ratio can be maintained at ∼1 across the tested conditions, suggesting excellent selectivity control. Therefore, a total GHSV of 192,000 mL h^−1^ g_cat_^−1^ (Ar flow rate as 22 mL min^−1^) is selected for subsequent tests.

Afterward, the influence of irradiance was investigated, as shown in Figure 1b. The surface temperature of Rh_2.5_/MnO_x_ increases with rising irradiance, from 229°C at 2.8 W cm^−2^ to 545°C at 12.8 W cm^−2^. As a result, the production rates of H_2_ and CO increase accordingly, reaching 952 and 990 mmol g^−1^ h^−1^ from initial values of 88 and 91 mmol g^−1^ h^−1^, respectively. Meanwhile, the CH_4_ and CO_2_ conversions increase from 6.4 and 6.4% to 51.8 and 51.9% over the same temperature range (Figure S7). Further increasing the irradiance from 12.8 W cm^−2^ to 14.6 W cm^−2^ only results in a moderate increase in both H_2_/CO production rates and CH_4_/CO_2_ conversions. To prevent excessive use of the Xenon lamp, an irradiance of 12.8 W cm^−2^ is selected for further performance tests. Moreover, the apparent activation energy (E_a_) of CH_4_ over Rh_2.5_/MnO_x_ is determined as 24.1 kJ mol^−1^ by the Arrhenius plot (Figure S8). This value is substantially lower than analogous Rh‐based photothermo catalysts (e.g., 42.8 kJ mol^−1^ for Rh/SrTiO_3_ [11]) and thermocatalysts (e.g., 62.3 kJ mol^−1^ for Rh/CeVO_3_ [60] and 65.9 kJ mol^−1^ for Rh/Al_2_O_3_‐La_2_O_3_ [61]) in the DRM reaction. This comparison indicates that MnO_x_ plays a unique role in facilitating the performance of Rh, as discussed later.

Therefore, the optimized experimental condition is a total GHSV of 192,000 mL h^−1^ g_cat_^−1^ with a CH_4_/CO_2_/N_2_/Ar composition of 4/4/2/22 and an irradiance of 12.8 W cm^−2^. To confirm the reproducibility of this photothermo catalyst, three Rh_2.5_/MnO_x_ samples were synthesized in three different batches and tested under light irradiation (Figures S9 and S10). The H_2_ and CO production rates (∼948 and ∼992 mmol g^−1^ h^−1^), together with H_2_/CO ratio (∼0.96) and CH_4_/CO_2_ conversions (∼51.9% and ∼52.5%), exhibit consistent values across the three samples, indicating the high reproducibility of this synthesis method.

Then, a long‐term stability test was conducted under the optimized conditions (Figure 1c). The light‐driven DRM performance remains stable during the 502‐h test on the Rh_2.5_/MnO_x_ catalyst despite slight fluctuations in gas flow. Because coke formation is a major cause of catalyst deactivation in DRM, its presence was examined using Raman spectroscopy. As shown in Figures S11 and S12, no characteristic peaks, such as those at 1350 cm^−1^ (D mode) and 1600 cm^−1^ (G mode) [62], are observed either at the beginning of the reaction (taken after 40 min time‐on‐stream) or after the long‐term stability test. In addition, O_2_‐TPO‐MS was performed on the spent catalyst after 30 h of reaction to evaluate the coke resistance of Rh_2.5_/MnO_x_ (Figure S13). The TPO‐MS profile shows only a weak and broad CO_2_ signal centred at around 275°C. This signal originates from trace adsorbed carbonaceous decomposition products, which can be consumed at relatively mild conditions, for example, our reaction process [63]. Importantly, no pronounced high‐temperature CO_2_ evolution peak is observed, indicating that no stubborn graphitic or filamentous coke, generally considered as a major cause of catalyst deactivation, is formed during the DRM reaction [64]. Furthermore, the carbon balance during the 502‐h stability test is presented in Figure S14. The carbon balance remains above 90% throughout the long‐term time‐on‐stream. These results indicate the exceptional coke resistance of the Rh_2.5_/MnO_x_ catalyst.

Given the nearly stoichiometric H_2_/CO ratio (∼1) observed during the 502‐h stability test, the CO production rate at the final run of the stability test period is used for comparison with reported photothermo catalytic DRM and thermocatalytic DRM (Figures 1d and S15). This comparison is intended to provide a contextual performance benchmark rather than a fully normalized performance comparison, because the reported systems in Figure 1d differ in catalyst mass, reactor geometry, GHSV, irradiation spectrum, and heating configuration (Note: the detailed reaction conditions for the benchmarked systems are summarized in Tables S2 and S3). The catalysts are compared under irradiance levels of a similar order of magnitude, which correspond to a temperature range of 350–580°C. The production rate of Rh_2.5_/MnO_x_ represents the highest reported value among both light‐driven and thermally driven DRM systems operating within a similar temperature range (< 600°C), advancing the production rate from the 0.1 mol g^−1^ h^−1^ regime to nearly 1 mol g^−1^ h^−1^. This corresponds to a real‐time syngas output of nearly 10 mmol h^−1^ without any catalyst‐weight normalisation, which already surpasses the performance of most light‐driven DRM systems [3, 7, 9, 11, 12, 13, 14, 18, 19, 20, 21, 22, 23, 26, 49, 50]. Furthermore, unlike a few catalysts (catalysts 4, 5, 8, and 16 in Figure 1d) that achieved CO production rates slightly above 0.5 mol g^−1^ h^−1^ only at the expense of low H_2_/CO ratios (0.45‐0.84), Rh_2.5_/MnO_x_ maintains a nearly stoichiometric H_2_/CO ratio of 0.96. Moreover, most photothermo DRM catalysts sustained activity for 5 to 150 h. Only a few photothermo catalysts, such as Ni/CeO_2_ [14] (catalyst 9 in Figure 1d) and Ni/CeO_2_ [10] (catalyst 2 in Figure 1d), have achieved extended stability longer than 200 h. However, their CO production rates are 16.6 and 8.7 times lower than that of Rh_2.5_/MnO_x_. Most reported thermocatalytic DRM catalysts exhibited stability for less than 40 h, more than one order of magnitude shorter than that of Rh_2.5_/MnO_x_. Only few thermocatalysts maintained activity for 100 h, such as Ni/CeO_2_ [51] (catalyst 16 in Figure 1d) and NiZnC/Al_2_O_3_ [55] (catalyst 21 in Figure 1d). These catalysts remain far less stable than Rh_2.5_/MnO_x_, even though their CO production rates are already lower (approximately 2‐fold and 4.5‐fold, respectively). Systems under low irradiance are also summarized in Table S4 for reference. Such comparison with benchmarks highlights the superiority of the activity and stability of Rh_2.5_/MnO_x_. Furthermore, considering the difficulty of fully normalising comparisons across different catalytic systems, more standardized benchmarking protocols are needed in future studies to enable more rigorous and fair evaluation of photothermo DRM performance.

Considering the intermittent nature of solar energy, intermittent stability is equally important for evaluating the practical applicability of the catalyst. Therefore, an intermittent stability test was designed, involving initiation of the DRM reaction, shutdown, air exposure, and subsequent resumption of the DRM reaction (Figure S16a). In this experiment, the Rh_2.5_/MnO_x_ catalyst is first evaluated under DRM conditions for 10 h under light irradiation, during which it shows stable performance. Subsequently, the light irradiation was switched off, and the catalyst bed was exposed to ambient air at room temperature for 12 h to simulate an overnight light‐off period. After switching the light irradiation back on and restarting the DRM reaction, the catalytic activity recovers to its original level and remains stable during the subsequent test. The structure of the catalyst also remains intact (Figure S16b).

Furthermore, the light‐to‐chemical efficiency was obtained to compare with previously reported light‐driven DRM catalysts with irradiance at a similar order of magnitude (Note: a high GHSV was applied to maintain low CH_4_ and CO_2_ conversions to minimise kinetic limitations and accurately reflect the intrinsic light‐responsive properties of the catalyst [65, 66]; detailed reaction conditions are provided in the Supporting Information). As shown in Figure S17, Rh_2.5_/MnO_x_ achieves a light‐to‐chemical efficiency of 29.5%, setting a new record among reported light‐driven DRM systems. In particular, compared to Rh/SrTiO_3_ (1.30%) [11] and CoNiRuRhPd/SrTiO_3_ (2.10%) [9], both of which contained Rh species as the catalytic centre but employed different supports, this work achieves an order of magnitude improvement in light‐to‐chemical efficiency. This clearly underscores the effective utilisation of photons across the full solar spectrum enabled by the unique MnO_x_ support.

To determine the origin of CO in the DRM reaction, isotope‐labelling experiments were performed (Figure S18). When ^12^CH_4_ and ^12^CO_2_ are used, a peak at *m/z*  =  28 appears, corresponding to ^12^CO. In contrast, using ^13^C‐labelled reactants (^13^CH_4_ and ^13^CO_2_) as the feeding gases, the *m/z* of carbon monoxide appears as a distinct signal at 29 in the mass spectrum. This can be assigned to ^13^CO, confirming that the carbon source is from the reactants rather than the impurities in the catalyst. To further resolve the contribution from CH_4_/CO_2_, mixed isotopic feeds of ^13^CH_4_/^12^CO_2_ and ^12^CH_4_/^13^CO_2_ are used. The resulting signal intensities of ^13^CO and ^12^CO are comparable, with an approximate 1:1 ratio. Collectively, these results confirm that CO originates from both CH_4_ and CO_2_ in the DRM process. Moreover, this observation further supports the stoichiometric nature of the DRM reaction, where the consumption of CH_4_ and CO_2_ occurs in an approximately 1:1 ratio, effectively suppressing side reactions, such as RWGS, CH_4_ decomposition, and CO disproportionation.

### 2.2 Structural Characterisations of the Catalysts

Figure 2a summarises the structural and chemical evolution of Rh_2.5_/MnO_x_ from the precursor to the final steady state. In particular, a clear transformation from Rh clusters to Rh nanoparticles, accompanied by the phase transition of Mn_2_O_3_ to MnO, is observed, as detailed below.

**FIGURE 2:**
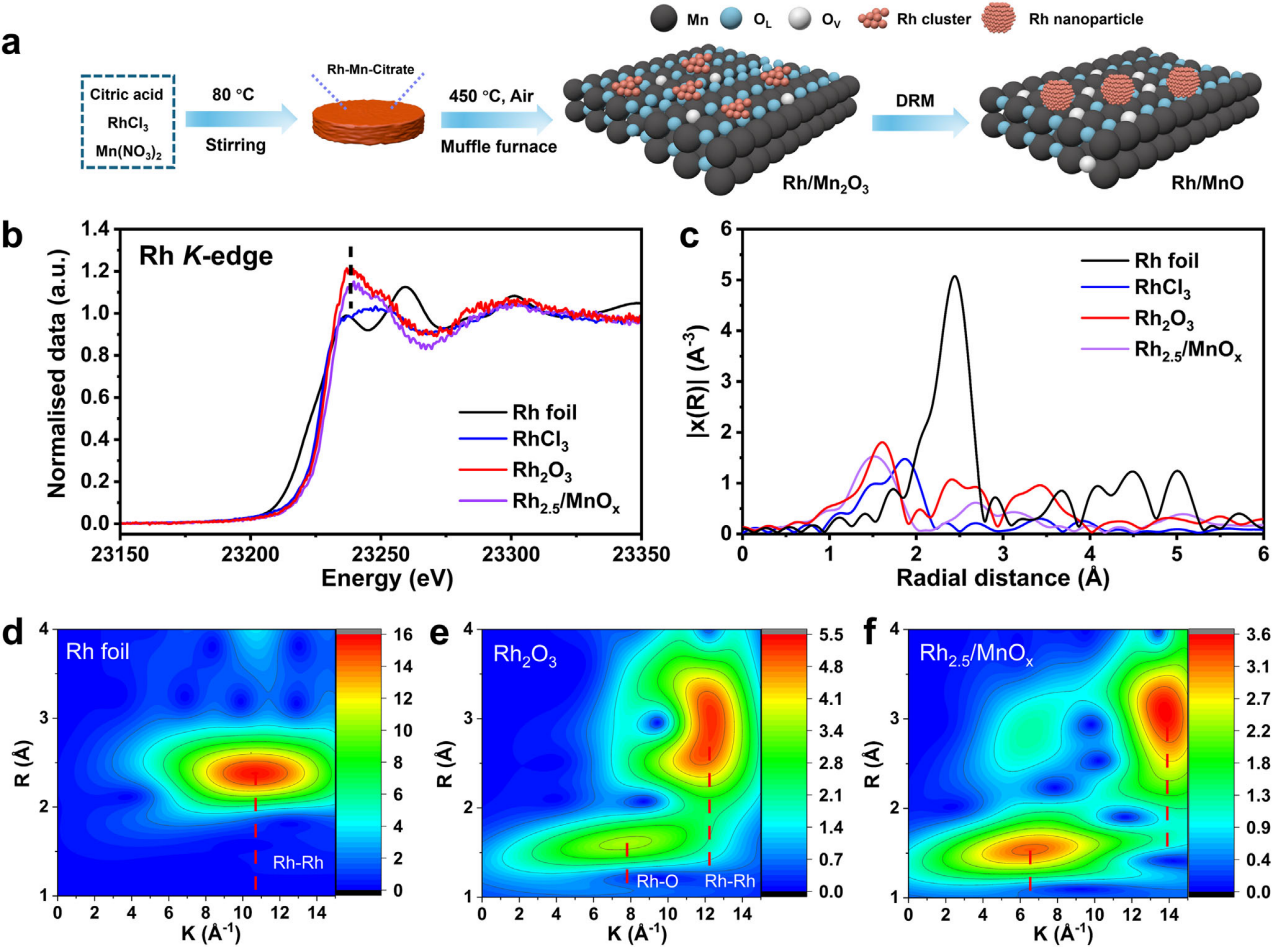
Structure characterisation and analysis of Rh_2.5_/MnO_x_. (a) Synthesis and transformation of Rh/MnO_x_ during catalyst preparation and DRM reaction. (b) Normalized Rh K‐edge XANES spectra of standard Rh foil, RhCl_3_, Rh_2_O_3_ and Rh_2.5_/MnO_x_. (c) k^2^‐weighted Fourier‐transform of the Rh K‐edge EXAFS spectra for standard Rh foil, RhCl_3_, Rh_2_O_3_ and Rh_2.5_/MnO_x_. (d–f) Wavelet‐transformed EXAFS spectra of standard Rh foil, Rh_2_O_3_ and Rh_2.5_/MnO_x_.

The sample after synthesis was investigated by high‐angle annular dark‐field scanning transmission electron microscopy (HAADF‐STEM) (Figure S19). As shown in Figure S19a,b, the support displays clear, distinct lattice fringe spacings of 0.27 nm, which correspond to the (222) planes of Mn_2_O_3_ [67]. Moreover, bright spots (∼1.4 nm) with higher contrast than the Mn_2_O_3_ support are uniformly distributed (Figure S19a), corresponding to Rh clusters. To further confirm their presence, line profiles are shown in two regions, with and without bright spots (Figure S19c,d). Intensified peaks corresponding to Rh species are evident in the central range of line 1 (Figure S19c), whereas they are absent in line 2 (Figure S19d), confirming the presence of Rh clusters on MnO_x_.

X‐ray absorption spectroscopy (XAS) was carried out to gain insights into the chemical state and local coordination environment of Rh in Rh_2.5_/MnO_x_ (Figure 2b–f). The pre‐edge absorption of Rh_2.5_/MnO_x_ closely resembles that of Rh_2_O_3_ and is higher than that of Rh foil, suggesting a Rh oxidation state of +3 (Figure 2b). The k^2^‐weighted Rh K‐edge extended X‐ray absorption fine structure (EXAFS) spectra of Rh_2.5_/MnO_x_, Rh foil, RhCl_3_ and Rh_2_O_3_ were compared in Figure 2c. The first‐shell scattering at ∼1.5 Å in R‐space for Rh_2.5_/MnO_x_ is similar to Rh_2_O_3_, while the absence of Rh‐Rh peaks and Rh‐O‐Rh peaks further supports the high dispersion of Rh. Compared with standard Rh foil (Figure 2d) and Rh_2_O_3_ (Figure 2e), the wavelet‐transformed EXAFS spectrum of Rh_2.5_/MnO_x_ exhibits two intensity maxima corresponding to Rh‐O and Rh‐Rh interactions (Figure 2f), closely resembling Rh_2_O_3_ and deviating significantly from Rh foil. Furthermore, Rh K‐edge EXAFS fitting reveals that Rh is primarily coordinated with oxygen atoms, comprising two Rh‐O shells with bond lengths of ∼1.95 and ∼2.09 Å, giving a total Rh‐O coordination number of 4.42 ± 1.5 (Figure S20 and Table S5). This coordination number is much smaller than that in Rh_2_O_3_, confirming the isolation and dispersion of Rh clusters on the MnO_x_ support.

Powder X‐ray diffraction (PXRD) was further used to investigate the structure before and after reaction (Figure 3a). Rh_2.5_/MnO_x_ before reaction shows characteristic diffraction peaks of Mn_2_O_3_ at 10.6°, 15.0°, 17.3°, 20.4°, 22.16°, 24.6° and 29.62°, which are associated with the (211), (222), (400), (332), (431), (440) and (631) planes, respectively [68, 69]. No peak assigned to Rh species can be observed, suggesting that Rh is highly dispersed in the form of small clusters on the MnO_x_ surface. This is consistent with the above STEM and XAS analysis. Interestingly, after 40 min time‐on‐stream, the XRD pattern of Rh_2.5_/MnO_x_ shows characteristic diffraction peaks of MnO at 15.9°, 18.4°, 26.1°, 30.7° and 32.1°, which are associated with the (111), (200), (220), (311) and (222) planes, respectively [70, 71]. Additionally, PXRD patterns were recorded at various reaction intervals to examine the timescale of structural reconstruction (Figure S21). Before the DRM reaction, MnO_x_ predominantly exists as Mn_2_O_3_. Once the reaction begins, Mn_2_O_3_ is rapidly reduced to MnO within 0.5 min, after which the MnO phase remains stable throughout the reaction. This transformation is attributed to the strongly reducing atmosphere created under the DRM conditions [8]. A similar transformation is also observed for pure MnO_x_ in the absence of Rh loading (Figure S22).

**FIGURE 3:**
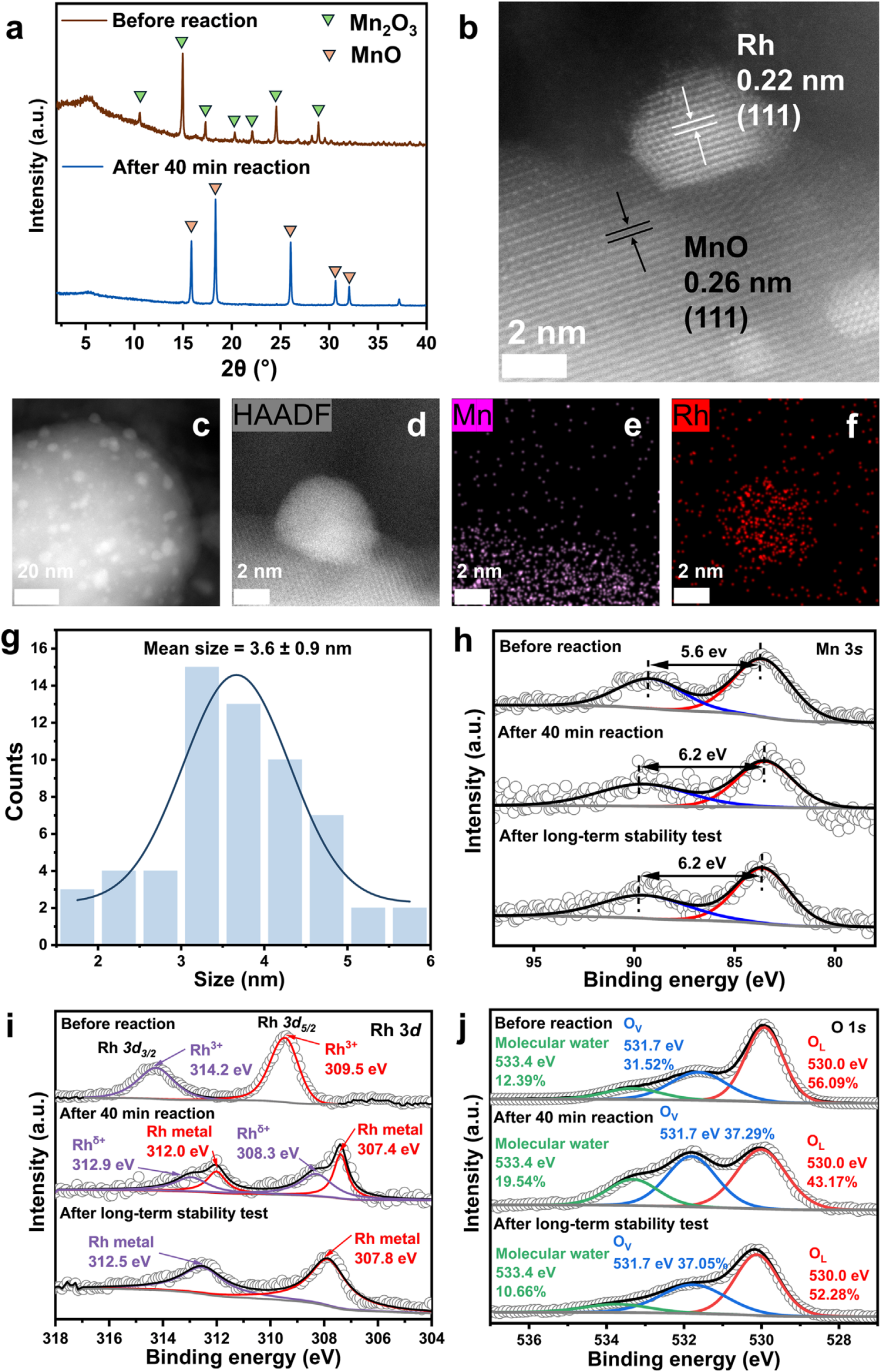
Structure characterisation and analysis of Rh_2.5_/MnO_x_. (a) PXRD spectra of Rh_2.5_/MnO_x_ before and after 40 min reaction. (b) High‐resolution HAADF‐STEM image showing Rh nanoparticles supported on MnO_x_ support after 40 min reaction. (c) HAADF‐STEM image of Rh_2.5_/MnO_x_ after 40 min reaction. (d‐f) High‐resolution HAADF‐STEM and EDX images of Rh_2.5_/MnO_x_ after 40 min reaction. (g) Particle size distribution of Rh nanoparticles (*Note*: 60 particles were counted. The fitting curve in Figure 3g was generated using a Gauss model). (h) Mn 3*s*, (i) Rh 3*d* and (j) O 1*s* XPS spectra of Rh_2.5_/MnO_x_ (taken before reaction, after 40 min reaction and after long‐term stability test).

HAADF‐STEM was conducted to examine the catalyst morphology after the DRM reaction. In clear contrast to the pre‐reaction state, bright nanoparticles appear uniformly on the support after 40 min of reaction (Figure 3b,c). The nanoparticles and support exhibit distinct lattice fringe spacings of 0.22 nm and 0.26 nm, corresponding to the (111) planes of Rh and MnO, respectively (Figure 3b) [72, 73]. The energy‐dispersive X‐ray spectroscopy (EDS) elemental mapping (Figure 3d–f) further confirms this assignment. The average size of the Rh nanoparticles is determined to be 3.6 ± 0.9 nm (Figure 3g). After a long‐term stability test (>500 h), the overall morphology of the spent Rh_2.5_/MnO_x_ is almost unchanged (Figure S23). Distinct lattice fringe spacings of 0.22 nm and 0.26 nm, corresponding to the (111) planes of Rh and MnO, respectively, can be clearly observed (Figure S23b) [72, 73]. EDS elemental mapping of Rh_2.5_/MnO_x_ after the long‐term stability test confirms that Rh nanoparticles are still uniformly dispersed on the MnO surface (Figure S23c–e). Furthermore, only a slight growth of Rh nanoparticles is observed from an average size of 3.6 nm to 4.9 nm (Figure S23f). Such structural stability and the absence of noticeable coking/sintering are considered key factors contributing to the sustained high catalytic activity of Rh_2.5_/MnO_x_ during long‐term operation.

XPS was carried out to reveal the chemical states of Mn, Rh, and O species during the structural transformation, as shown in Figure 3h–j. Since the peaks of MnO and Mn_2_O_3_ often overlap in the Mn 2*p* region [74, 75], Mn 3*s* is used instead to explore the oxidation state of Mn in Rh_2.5_/MnO_x_ [76]. Mn 3*s* typically exhibits two multiplet split peaks arising from exchange coupling [77], and the energy separation (Δ*E_s_*) between these two peaks is widely used to estimate the actual average oxidation state (*AOS*) of surface Mn using the formula given below [78]:

$$AOS=8.956-1.126\times \Delta E_{s}$$

The Rh_2.5_/MnO_x_ before reaction shows an energy separation of 5.6 eV, while the spent samples both exhibit an increased Δ*E_s_* of around 6.2 eV (Figure 3h). As a result, the *AOS* of surface Mn in Rh_2.5_/MnO_x_ sample before and after reaction can be calculated as 2.65 and 1.97. It is interesting to read that Mn^2.65+^ is lower than that of the Mn^3+^ in Mn_2_O_3_ obtained by PXRD and STEM analysis, this indicates the presence of O_V_ as discussed below. In contrast, after 40 min reaction, the valence state has been reduced to 1.97, which is close to +2, consistent with the PXRD and STEM analysis above. Again, the slight deviation of valence state suggests the presence of O_V_. The *AOS* value of Rh_2.5_/MnO_x_ after long‐term reaction is identical to that after 40 min reaction, suggesting that the structure remains stable during the DRM reaction after the initial transformation. In the Rh 3*d* XPS spectra, the fresh Rh_2.5_/MnO_x_ sample mainly exhibits Rh^3+^ species, with the Rh 3*d*_5/2_ and Rh 3*d*_3/2_ peaks at 309.5 eV and 314.2 eV, respectively (Figure 3i) [79]. After 40 min DRM reaction, the Rh 3*d* region can be deconvoluted into two distinct Rh states. The first component is assigned to metallic Rh^0^, showing characteristic peaks at 307.4 eV for Rh 3*d*_5/2_ and 312.0 eV for Rh 3*d*_3/2_ [80]. The second component is attributed to partially oxidized Rh species, with the Rh 3*d*_5/2_ and Rh 3*d*_3/2_ peaks appearing at 308.3 eV and 312.9 eV, respectively [81]. These results indicate that the Rh species undergo reduction during the reaction, consistent with the Rh nanoparticles formation observed via STEM. Notably, after the long‐term stability test, only metallic Rh^0^ (3*d*_5/2_: 307.8 eV, 3*d*_3/2_: 312.5 eV) is detected, indicating that Rh species have been completely reduced under the reaction conditions [82]. Compared with the sample collected after 40 min reaction, a slight positive shift in the binding energy for the metallic Rh species is observed after long‐term stability test, which may be attributed to the adsorption of CO on Rh nanoparticles during the DRM reaction [83]. The O 1*s* XPS spectra were further conducted to analyse the O_L_ and O_V_ (Figure 3j). The peak at 530.0 eV is assigned to O_L_, whereas the component centred at 531.7 eV is attributed to defect‐related oxygen species (e.g., surface OH^−^/O_2_), which are usually associated with O_V_ [84, 85, 86]. In addition, the weak peak located at 533.4 eV is surface‐adsorbed molecular water [86]. Interestingly, after 40 min reaction, the O_V_/O_L_ ratio increases from 0.56 to 0.85, indicating a substantial O_V_ presence after the structural transformation from Mn_2_O_3_ to MnO. Following the long‐term stability test, the ratio decreases to 0.71, likely due to partial O_L_ replenishment by CO_2_ during the reaction. It should be noted that this value remains higher than the initial 0.56, suggesting the preservation of relatively high and stabilized O_V_ density throughout the dynamic O_L_‐O_V_ cycle under reaction conditions. As the O 1*s* component associated with effective O_V_ in XPS may also contain contributions from surface OH^−^/O_2_, the O_V_/O_L_ ratio is used only as a supporting reference. The O_V_ formation and its participation in the O_L_‐O_V_ cycle are further verified by EPR and the subsequent ^18^O‐labelling experiment in the later section.

To provide direct evidence of the O_V_ presence, Electron Paramagnetic Resonance (EPR) measurements were conducted on the activated Rh_2.5_/MnO_x_ samples. As shown in Figure S24, the EPR spectrum of Rh_2.5_/MnO_x_ after the DRM reaction exhibits a symmetric signal in the g ≈ 2.004 region. This signal is a characteristic fingerprint of unpaired electrons trapped at O_V_, indicating the presence of O_V_ in Rh_2.5_/MnO_x_ after the DRM reaction [8]. This EPR observation is in agreement with the XPS results presented in Figure 3j.

All the above results suggest that the Rh_2.5_/MnO_x_ catalyst undergoes an immediate structural transformation during the DRM reaction, in which Mn_2_O_3_ is rapidly converted to MnO with the preservation of abundant O_V_. Meanwhile, the oxidized Rh clusters gradually evolve into metallic Rh nanoparticles. This evolved structure is stabilized as the dominant configuration of the catalyst under the steady state.

### 2.3 Investigation of Photon‐to‐Phonon Pathway

The light‐harvesting abilities were investigated using ultraviolet‐visible‐near‐infrared diffuse reflectance absorption spectroscopy (Figure 4a). The as‐synthesized MnO_x_ exhibits broadband absorption across 200–2500 nm, indicating strong potential as an efficient solar light harvester. This absorption profile closely resembles that of reported Mn_2_O_3_ [27], consistent with the above structural analysis. Negligible changes can be observed upon Rh incorporation, likely due to its low loading amount and highly dispersed cluster state. After 40 min of DRM reaction, the MnO_x_ absorption spectrum shows reduced intensity beyond 285 nm compared with Mn_2_O_3_, attributable to the structural transformation to MnO discussed earlier [30]. Interestingly, unlike typical MnO, which often exhibits dominant UV absorption and poor visible light response [33, 87], the post‐reaction MnO_x_ displays extended absorption up to 2500 nm, due to the defect levels induced by the O_V_ [35, 36, 37]. Additionally, the Rh_2.5_/MnO_x_ after reaction shows stronger absorption above 500 nm than bare MnO_x_ after reaction, which can be ascribed to light scattering from the formation of Rh nanoparticles [88]. Collectively, this result suggests that the MnO_x_ likely serves as the primary light harvester in the catalyst system.

**FIGURE 4:**
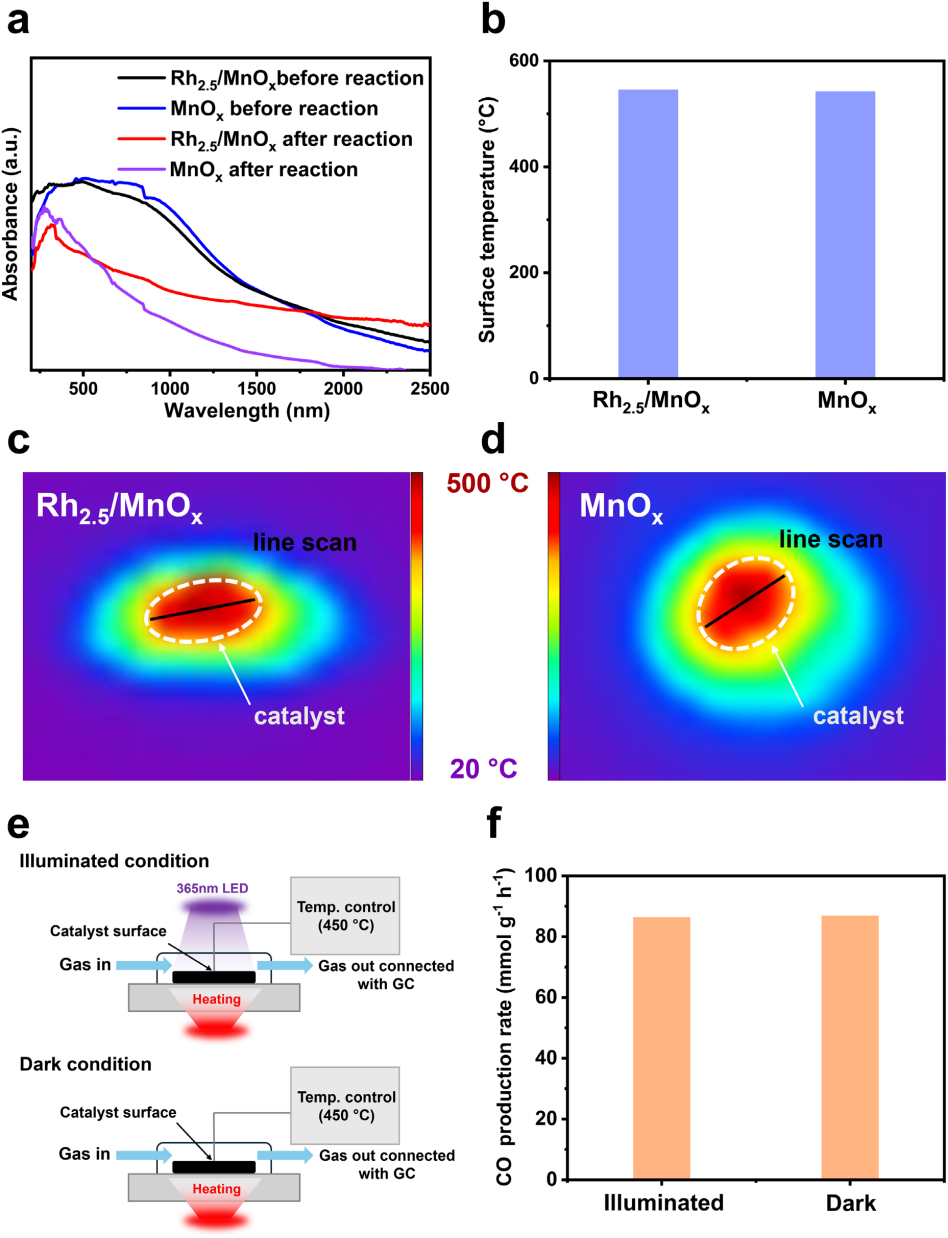
The mechanism of the photons‐to‐phonons channel. (a) Diffuse reflectance UV‐vis‐NIR absorption spectra of Rh_2.5_/MnO_x_ before reaction, MnO_x_ before reaction, Rh_2.5_/MnO_x_ after reaction, and MnO_x_ after reaction. (b) Surface temperature of Rh_2.5_/MnO_x_ and MnO_x_. Surface temperature monitoring of Rh_2.5_/MnO_x_ (c) and MnO_x_ (d) by using a thermal imaging camera. (Only the red region enclosed by the white circle corresponds to the catalyst with a diameter of 10 mm, and the light spot was confined solely to this area). (Reaction conditions: Figure 4b: 10 mg catalyst; CH_4_/CO_2_/N_2_/Ar = 4/4/2/22; GHSV = 192,000 mL h^−1^ g_cat_^−1^, light wavelength = 200–2500 nm; irradiance = 12.8 W cm^−2^; Figure 4c,d: the same as Figure 4b except the irradiance was changed to 9.8 W cm^−2^). (e) Schematic illustration of the experimental setup designed to decouple the photo‐thermo contribution in the DRM reaction. (f) CO production rates of Rh_2.5_/MnO_x_ catalyst with/without light irradiation at 450°C. (Illuminated condition: 1 mg catalyst, surface temperature = 450°C, external heating with 365 nm LED light, irradiance = 0.36 W cm^−2^; Dark condition: 1 mg catalyst, surface temperature = 450°C, external heating without light irradiation).

The surface temperature of Rh_2.5_/MnO_x_ and MnO_x_ during the reaction was monitored under identical light irradiation (12.8 W cm^−2^), as shown in Figure 4b. Both samples rapidly reach comparable surface temperatures of 545 and 541.8°C, respectively. To further examine the distribution of the localized thermal field, a thermal imaging camera was employed (Note: a lower irradiance was used to avoid exceeding the camera's maximum measurable temperature of 550°C), as presented in Figures 4c,d and S25. Both samples exhibit a uniform photo‐to‐thermo region (Figure 4c,d). A line‐scanning profile reveals a gradual temperature decrease from the centre to the edge under identical light irradiation (9.8 W cm^−2^), with similar temperature ranges of 465–498°C and 464–492°C for Rh_2.5_/MnO_x_ and MnO_x_, respectively (Figure S25). This small spatial temperature deviation ensures a homogeneous heating environment conductive to the DRM reaction. These results confirm MnO_x_ as the primary light absorber, capable of efficiently converting absorbed photons into phonons to generate a localized thermal field.

To further clarify the role of photons in the DRM reaction over Rh_2.5_/MnO_x_, a series of control experiments were conducted to decouple the photon‐to‐electrons/holes and photon‐to‐phonon pathways (Figure 4e). A 365 nm light source was selected because its photon energy matches the bandgap of MnO_x_ (Figure 4a), enabling electron‐hole excitation. To avoid underestimating the actual catalyst temperature under high‐irradiance illumination, which could lead to an overestimation of the photon‐enhanced effect [89], the irradiance was kept as low as 0.36 W cm^−2^. The reaction temperature was strictly controlled at 450°C under both illuminated and dark conditions. Using the CO production rate as a performance indicator, the results under both conditions are nearly identical (Figure 4f). This result indicates that photoexcited electron‐hole pairs play a negligible role in the DRM process. Therefore, the dominant mechanism is photon‐to‐phonon conversion, where absorbed photons in MnO_x_ primarily contribute to the generation of a localised thermal field. A comparative experiment under concentrated‐light and purely thermal conditions was designed to provide an insightful understanding of the localized thermal field at the micrometre scale (Figures S26–S27 and Note S2), which further confirms its capability to drive the DRM reaction.

### 2.4 Mechanistic Insights Into Surface Chemical Processes

The reactivity of O_L_ in MnO_x_ was first investigated using H_2_‐TPR profiles (Figure S28), with SiO_2_ and TiO_2_ as references. The MnO_x_ was pretreated under reaction atmosphere to reflect its actual state. SiO_2_ shows negligible reduction peaks within this temperature range due to its highly stable O_L_ [90]. In contrast, TiO_2_, often employed to generate O_V_ for enhanced substrate activation via reduction pretreatment in photocatalysis [91], exhibits a peak at 516°C. It should be noted that MnO_x_ displays a distinct reduction peak at 425°C, significantly lower than that of TiO_2_, indicating that O_L_ in MnO_x_ is more reactive. Moreover, the peak area of MnO_x_ is 4.4 times larger than that of TiO_2_, suggesting a substantially higher concentration of active O_L_ species. These characteristics are expected to play a critical role in the light‐driven DRM process through the dynamic O_L_‐O_V_ cycle [92].

Systematic in situ diffuse reflectance infrared Fourier transform spectroscopy (DRIFTS) experiments under switching gas atmospheres were designed to resolve the role of O_L_ and O_V_ during the DRM reaction. Because the catalysts undergo structural changes under DRM conditions, all samples were preactivated in a DRM reaction atmosphere to ensure the reflectance of real active centres. When CH_4_ was introduced individually, in addition to its characteristic asymmetric C─H stretching vibration (3009 cm^−1^) and C─H deformation vibration (1303 cm^−1^) (Figure S29a,b) [93, 94, 95], a band at 2006 cm^−1^ corresponding to linearly adsorbed CO on Rh is observed (Figure 5a) [96, 97]. This indicates that CH_4_ can be activated by O_L_ from the MnO_x_ support to form CO. The generation of CO_x_ species results in gradual O_L_ depletion and O_V_ formation. Next, Ar was purged through the cell to remove the residual gases in the system, and CO_2_ was then introduced individually. After adsorption, the temperature was raised under batch conditions. Interestingly, a new band at 2030 cm^−1^ appears (Figure 5b), assigned to multicarbonyl species adsorbed on Rh ((CO)_x_‐Rh) [79, 97]. This observation implies that upon CO_2_ introduction, the as‐formed O_V_ is refilled by abstracting oxygen from CO_2_, generating CO. It is likely that part of the newly formed O_L_ also consumes residual C* species left on Rh during the CH_4_ cycle. The static sealed cell thus creates a CO‐rich environment, favoring multicarbonyl adsorbed species. To verify whether the regenerated O_L_ retains its reactivity, CH_4_ was reintroduced after removing residual species from the CO_2_ cycle (Figures 5c and S29c,d). Similar to the first run, CO reappears at 2006 cm^−1^ upon heating (Figure 5c), confirming successful recovery of reactive O_L_.

**FIGURE 5:**
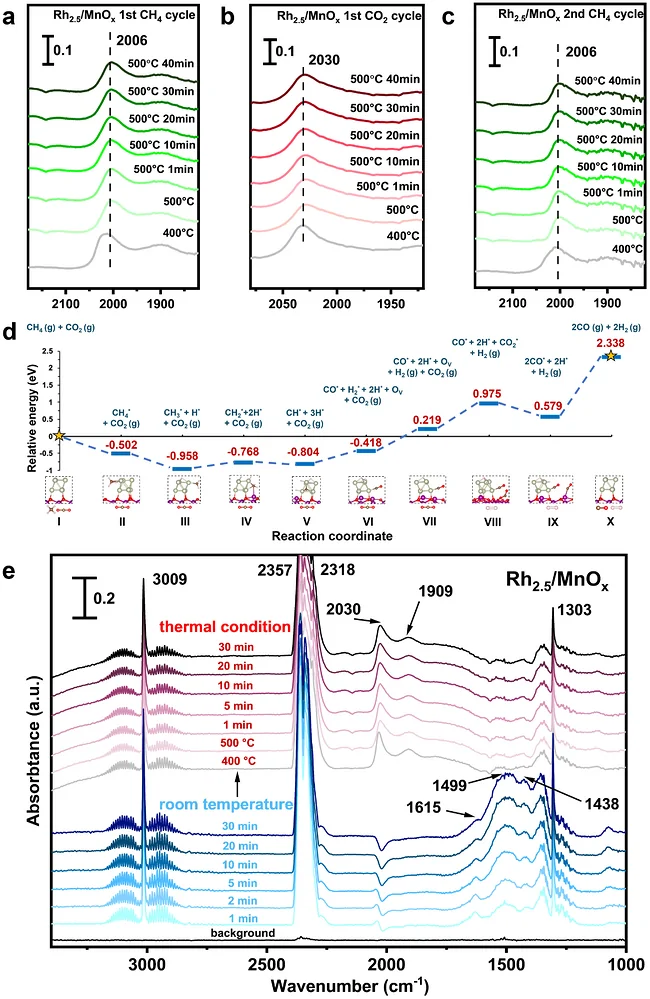
Surface chemistry of the Rh_2.5_/MnO_x_ interface. (a–c) In situ DRIFTS spectra (switching gas mode) over Rh_2.5_/MnO_x_. (d) Thermodynamic reaction‐energy profile of the entire DRM reaction pathway on the Rh/MnO (111) catalyst system, determined via DFT (all species with * are adsorbed species). (e) In situ DRIFTS spectra for DRM over Rh_2.5_/MnO_x_ under thermal conditions (Reaction condition: 10 mg catalyst, CH_4_/CO_2_/Ar = 4/4/22; GHSV = 180,000 mL h^−1^ g_cat_^−1^, temperature = room temperature‐500°C).

To confirm the O_V_ is generated and participates in CO_2_ activation, a purposefully designed ^18^O‐labelling experiment was conducted. A certain amount of Rh_2.5_/MnO_x_ catalyst was loaded into a home‐made flow reactor. The reactor could be switched to batch mode by closing the valves at both ends. The catalyst was first exposed to CH_4_ and C^16^O_2_ for 120 min, allowing surface reconstruction under reaction conditions and establishing surface O_L_ dominated by ^16^O active species. The MS spectrum of CO only shows a single peak corresponding to C^16^O (*m*/*z* = 28), as shown in Figure S30. After high‐temperature Ar purging to remove adsorbed species, a CH_4_ and C^18^O_2_ mixture was introduced to fill the reactor. Under a batch‐mode reaction for 2 min, apart from the formation of C^18^O (*m*/*z* = 30), a clear signal corresponding to C^16^O (*m*/*z* = 28) is also detected (Figure S31a). Since the oxygen source in the gas phase is C^18^O_2_, the appearance of C^16^O indicates that carbon from CH_4_ reacts directly with ^16^O_L_ on the catalyst surface to produce C^16^O. After the first cycle, the catalyst was purged with Ar under high‐temperature conditions again and then exposed to a CH_4_ and C^16^O_2_ mixture for the second batch‐mode reaction (Figure S31b). A detectable C^18^O signal (*m/z* = 30) is still observed. This result indicates that ^18^O from C^18^O_2_ in the first cycle had been incorporated into the catalyst lattice and subsequently participated in the CH_4_ activation to form C^18^O during the second cycle. In other words, during the methane activation process, O_L_ is consumed, leading to the formation of O_V_, which actively participates in CO_2_ activation in the DRM reaction. Therefore, these results, together with the DRIFTS experiments under switching gas atmospheres, show that MnO_x_ provides a dynamic O_L_‐O_V_ cycle that removes surface carbon species from CH_4_ dehydrogenation and activates CO_2_, thereby sustaining the stable and efficient DRM catalytic cycle. The superiority of the as‐prepared MnO_x_ support was further investigated by comparison with commercial MnO and Mn_2_O_3_ supports (Figures S32–S36 and Note S3).

To confirm the indispensable role of Rh in the DRM reaction, similar in situ DRIFTS experiments under switching gas atmosphere were conducted on pure MnO_x_ (Figure S37a–c). Notably, no bands corresponding to CO are detected during the CH_4_ cycles (Figures S37a and S37c), indicating that CH_4_ activation via the O_L_ requires Rh, consistent with its well‐established ability to cleave inert C‐H bonds and initiate the catalytic cycle [11, 98]. The absence of CO in the first CH_4_ cycle further suggests that O_V_ are not generated at this stage. During the CO_2_ cycle, no CO formation is observed (Figure S37b), confirming that without O_V_, CO_2_ activation cannot occur. These findings align with the performance data shown in Figure S2.

To elucidate the synergistic effect between Rh and MnO_x_ support during the light‐driven DRM reaction, systematic DFT simulations were conducted for the catalyst structure at steady state (Figures 5d and S38–S42 and Tables S6–S9). Firstly, CH_4_ adsorption configurations were investigated on two Rh/MnO models: the Rh/MnO (110) system (Figure S38 and Table S6) and the Rh/MnO (111) system (Figure S39 and Table S7). Three different CH_4_ adsorption site types are identified: cluster sites (adsorption solely onto the Rh cluster), interface sites (adsorption at the interface between the Rh cluster and MnO surface), and support sites (adsorption solely on the MnO surface). As shown in Figure S38 and Table S6, the most favorable CH_4_ adsorption site (site 1a) over Rh/MnO (110) is on the Rh cluster, exhibiting the lowest adsorption energy of −0.543 eV. Similarly, for the Rh/MnO (111) system, the most favorable adsorption site is again located on the Rh cluster, with a comparable adsorption energy of −0.502 eV (site 1b, as shown in Figure S39 and Table S7). Subsequently, Rh/MnO (111) was chosen as the model catalyst on which to investigate the entire DRM process, as this facet is observed in the HAADF‐STEM images in Figure 3b.

For each reaction step of the DRM process, several different product geometries were created, and their DFT energies were compared (detailed information on each product geometry was provided in Figure S40 and Table S8). The most energetically favorable geometry for each reaction step is then used to construct the stepwise optimal reaction pathway and the corresponding thermodynamic reaction energy profile for the entire DRM process (Figure 5d). Step I corresponds to the clean Rh/MnO (111) catalyst, while Step II represents the aforementioned lowest‐energy CH_4_ adsorption configuration (−0.502 eV). Upon the adsorption of CH_4_ to Rh/MnO (from Step I to Step II), an elongation of C─H bonds can be observed (Figure S41 and Table S9), suggesting the deformation of CH_4_ due to pre‐activation by Rh. The pre‐activated CH_4_ then experiences C─H bond cleavage to form CH_3_* with a favorable reduction in relative energy of 0.456 eV (Step III), where the as‐formed CH_3_* is preferentially stabilized on the Rh cluster (Figure S40 and Table S8). No interaction is observed between O_L_ and CH_3_* for the products in Step III indicating that CH_3_* tends to undergo further dissociation. CH_3_* is dissociated to CH_2_* which again preferentially resides on the Rh cluster (Step IV). It is interesting to find that Mn atoms at the cluster‐support interface may sometimes stabilise the CH_2_* radical (Figure S40). This interaction is likely owing to the carbide‐like interaction that is not uncommon during CH_4_ activation on transition‐metal‐based catalysts [99]. Further dissociation generates CH* (Step V), which is an energetically favorable process. As shown in Figure S40, CH* is still on Rh cluster since the structure with CH*‐O_L_ configuration shows a much higher energy (>1.8 eV). Thereafter, in Step VI, dissociation of the final C‐H bond yields a C*‐radical, and a strong C*‐O_L_ interaction is clearly established, resulting in the formation of an O_V_ together with CO*. Thus, the entire CH_4_ dissociation sequence proceeds most favorably on the Rh cluster, and the interaction of O_L_ and the carbon atom in CH_4_ only occurs after complete dissociation of the CH_4_ molecule. Furthermore, the as‐formed CO prefers to adsorb on Rh site, consistent with the analysis in in situ DRIFTS above. The formation of H_2_* by recombination of H* is also observed in this step. From Step VII to Step VIII, H_2_* is released as H_2_ gas, and the CO_2_ molecule can be adsorbed and preactivated at the O_V_. This ultimately leads to the formation of CO* and the regeneration of O_L_, accompanied by a favorable stepwise energy decrease of 0.396 eV (Step IX). The entire DRM cycle is then completed, followed by the release of CO and H_2_ as gaseous products in Step X. Additionally, the effect of additional O_V_ on the interaction between the Rh and the MnO support was investigated (Figure S42 and Note S4), which suggests that the presence of O_v_ near the Rh anchoring site may stabilise Rh nanoparticles against sintering. This result is consistent with the HAADF‐STEM observation that only slight growth of Rh nanoparticles was observed after a long‐term stability test in Figure S23f.

In situ DRIFTS under the simulated DRM reaction conditions was carried out to provide a full picture of the reaction (Figure 5e). At room temperature, the characteristic asymmetric C─H stretching vibration at 3009 cm^−1^ and the C─H deformation vibration at 1303 cm^−1^ attributable to CH_4_ [93, 94, 95], as well as the C═O stretching vibration of CO_2_ (2357 cm^−1^ and 2318 cm^−1^) [100, 101] are clearly observed. The vibration bands at 1615 cm^−1^ and 1499 cm^−1^ can be assigned to the asymmetric ν_as_(OCO) stretching modes of surface bidentate and monodentate carbonate species, while the corresponding symmetric ν_s_(OCO) stretching vibration of bicarbonate appears at 1438 cm^−1^ [102]. These features, together with the gradual increase in peak intensities, indicate the gradual adsorption of the reaction gases on the catalyst surface. When the temperature increases to 500°C, multicarbonyl species adsorbed on Rh (2030 cm^−1^, (CO)_x_‐Rh) [79, 97] and bridge‐adsorbed CO species (1909 cm^−1^, (Rh^0^)_x_‐CO) [79, 96] are immediately observed on Rh_2.5_/MnO_x_, suggesting fast CO generation and strong CO binding on Rh surfaces. The pronounced presence of (CO)_x_‐Rh suggests a high CO production rate and efficient turnover of the DRM cycle enabled by the rapid removal of carbon species and continuous regeneration of active sites. As the reaction proceeds for up to 30 min, no noticeable attenuation of the CO adsorption bands is observed, indicating that the O_L_‐O_V_ redox cycle on Rh_2.5_/MnO_x_ remains stable. Furthermore, the lack of COOH* (1716 cm^−1^) [102, 103, 104] and HCOO* (1550 cm^−1^) [38, 105] clearly distinguishes this system from hydrogen‐assisted CO_2_ dissociation mechanisms. This observation supports the O_V_‐driven CO_2_ dissociation pathway, which is consistent with the DFT results.

Based on the above results, a scheme for the light‐driven DRM reaction over Rh_2.5_/MnO_x_ is proposed (Scheme 1). Under full‐spectrum light irradiation, MnO_x_ absorbs photons via the intrinsic bandgap excitation and O_V_‐related defect level transitions and efficiently converts the absorbed energy into phonons by the recombination of charge carriers. This photon‐to‐phonon process induces a homogeneous local thermal field (∼545°C) rapidly, providing sufficient driving force for DRM. CH_4_ is activated on Rh sites, where successive C─H bond cleavage releases H_2_ and produces surface C* intermediates. O_L_ from MnO_x_ rapidly oxidises C* to form CO, thereby suppressing carbon accumulation and simultaneously creating O_V_. The generated O_V_ serves as the preferred adsorption and activation site for CO_2_, facilitating CO_2_ dissociation to produce additional CO and replenish the consumed O_L_. Through this interfacial O_L_‐O_V_ redox sequence, including CH_4_ activation, O_L_ consumption, O_V_ formation, CO_2_ activation, O_V_ replenishment, and O_L_ regeneration, an efficient and stable DRM reaction cycle is established.

**SCHEME 1:**
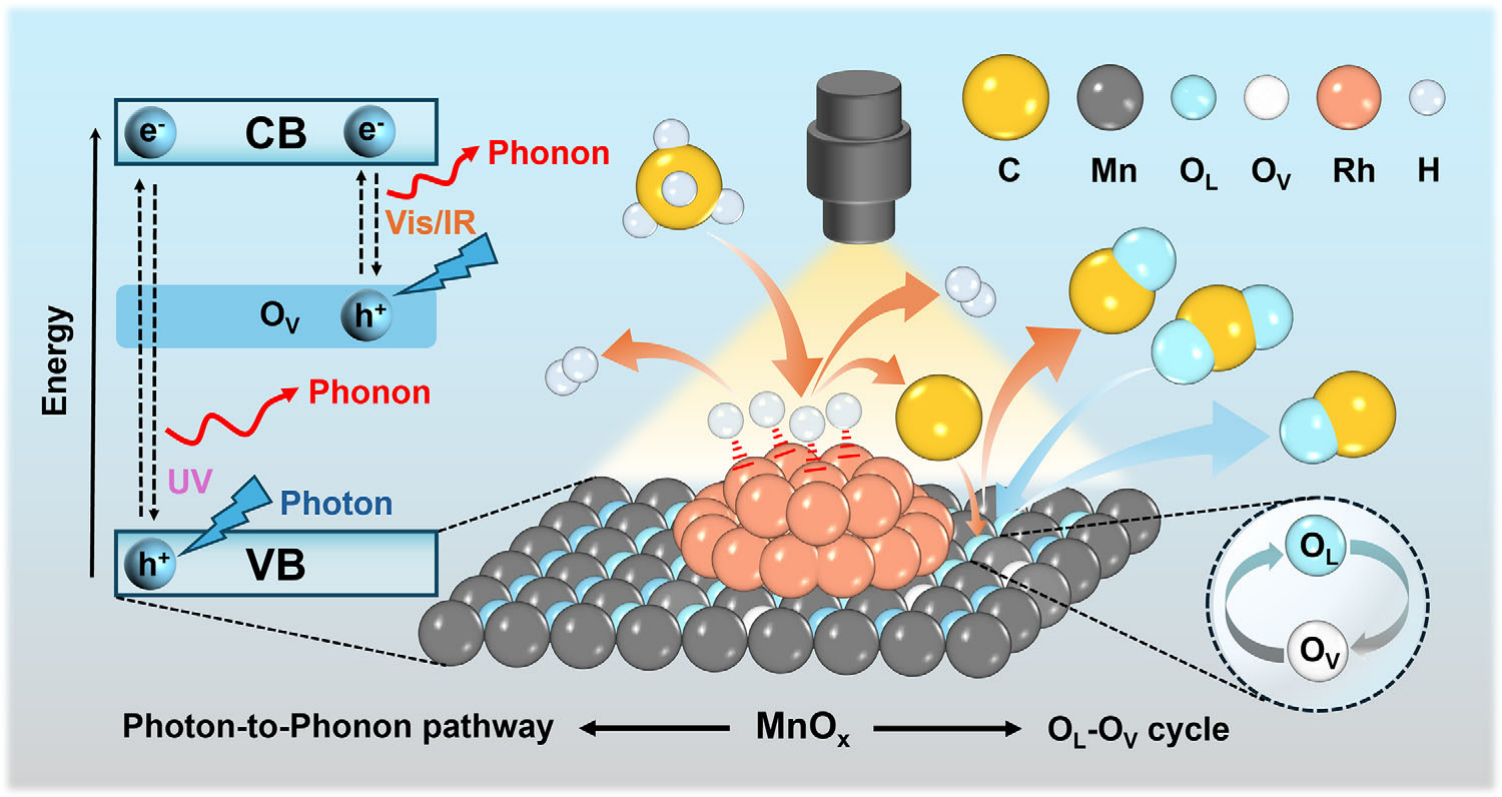
Proposed reaction scheme for light‐driven DRM over Rh_2.5_/MnO_x_.

Although the Rh_2.5_/MnO_x_ catalyst exhibits high performance in terms of syngas productivity, stability and light‐to‐chemical efficiency, it should be noted that the Rh loading (2.68 wt.%) remains relatively high. Given the scarcity and high cost of Rh as a noble metal, further optimisation of the active metal component is still desirable. Several strategies can be considered to address this issue. For example, since Rh is present as nanoparticles in the current Rh/MnO_x_ system, single‐atom engineering may provide an effective route to improving the atomic utilisation efficiency of Rh. This strategy has been widely explored in the direct conversion of methane to acetic acid, highlighting its potential for maximising noble metal efficiency in methane activation [106, 107]. In addition, alloying Rh with non‐noble metals can also be a promising strategy to reduce Rh usage while modulating its electronic structure and catalytic behavior. Successful examples of Rh‐based alloys, such as RhCu alloys in propane dehydrogenation [108] and RhFe alloys in CO_2_ hydrogenation to ethanol [109], have been reported. These strategies may be further integrated with the present MnO_x_ support platform to achieve more efficient methane conversion with reduced noble metal usage.

## 3 Conclusion

In summary, the photothermo catalyst Rh/MnO_x_ has been synthesized for syngas production via light‐driven DRM reaction. Record‐high stable syngas production rates (H_2_: 948 mmol g^−1^ h^−1^; CO: 992 mmol g^−1^ h^−1^) together with a near‐unity H_2_/CO ratio are achieved under full‐spectrum light irradiation. Exceptional stability is demonstrated by maintaining performance over 502 h with no detectable graphitic/filamentous coke accumulation under the Raman and TPO‐MS analyses. Furthermore, under a separate low‐conversion, high‐GHSV protocol, a light‐to‐chemical efficiency (29.5%) can also be reached. Fundamentally, this outstanding performance arises from two key aspects related to photophysics and surface chemistry enabled by the unique MnO_x_ support: (1) Acting as a broadband light harvester (up to ∼2500 nm) with an efficient photon‐to‐phonon conversion pathway, generating intense local thermal fields to meet kinetic and thermodynamic requirements of DRM; (2) Facilitating a dynamic O_L_‐O_V_ cycle, where active O_L_ consumes C* species formed from complete CH_4_ dehydrogenation on Rh sites, while O_V_ are generated for CO_2_ activation, thereby suppressing coke formation and promoting continuous catalytic turnover. This work establishes a new avenue for efficient solar energy utilisation and syngas synthesis by shifting the conventional focus from active‐site design to multifunctional support engineering.

## Author Contributions

Y. L. and X. L. designed the project and supervised the whole project. C. G. designed and conducted experiments, performed characterisations, and analysed data. X. L. and C. G. co‐drafted the article. A. J., S. J. and X. Z. carried out the DFT simulations and analysed the results. J. F. performed XAS experiments and contributed to the data analysis. X. L., X. L., J. W., L. C. and X. L. assisted with data analysis and mechanism discussions.

## Conflicts of Interest

The authors declare no conflicts of interest.

## Supporting information




**Supporting File**: anie73946‐sup‐0001‐SuppMat.docx.

## Data Availability

The data that support the findings of this study are available in the supplementary material of this article.
