# Supplementary material for: Synergy Between Photon‐to‐Phonon Pathway and Active Lattice Oxygen Enables Efficient and Stable Syngas Synthesis
Source: Angew Chem Int Ed Engl. 2026 Aug 5;65(40):e9236832. doi: 10.1002/anie.9236832 (PMC13618302; doi:10.1002/anie.9236832)
Supplement: Supplementary file 1 [file ANIE-65-e9236832-s001.docx]

**Supporting Information**

**Synergy between Photon-to-Phonon Pathway and Active Lattice Oxygen Enables Efficient and Stable Syngas Synthesis**

Chengzhi Guo,^[a]^ Apoorv Jain,^[b]^ Junrun Feng,^[c]^ Xinyu Li,^[a]^ Xinru Li,^[a,d]^ Shuya Jia,^[b]^ Juncong Wang,^[a]^ Leirun Chen,^[a]^ Xinjie Luo,^[a]^ Xiaolei Zhang,^[b]^ Xiyi Li,*^[a]^ and Yang Lan*^[a]^

[a]	Department of Chemical Engineering
University College London
Gower Street, London, WC1E 7JE, United Kingdom
E-mail: uceclii@ucl.ac.uk; yang.lan@ucl.ac.uk

[b]	Department of Chemical and Process Engineering
University of Strathclyde
Glasgow, UK

[c]	School of Science, School of Chip Industry
Hubei University of Technology
Wuhan, Hubei 430068, China

[d]	College of New Energy
Xi'an Shiyou University
Xi'an, Shaanxi 710065, China

**Methods**

**Synthesis of Rh/MnO_x_**. The Rh/MnO_x_ catalysts were prepared using the modified citric acid combustion method^[1]^. In a typical experiment, 0.5 mmol of Mn(NO_3_)_2_·4H_2_O (97%, Sigma-Aldrich), 0.5 mmol citric acid (99.5%, Fisher scientific) and different amounts of RhCl_3_·xH_2_O (38-40% Rh, Sigma-Aldrich) were mixed with tiny amount of deionized (DI) water (~1 mL) to produce a viscous gel under vigorous stirring and heating at 80 °C in an alumina crucible. The as-prepared gel was calcined at 450 °C for 3 h with the heating rate of 5 °C min^–1^. The samples were collected by grinding and denoted as Rh_a_/MnO_x_ (a=1, 2.5 and 5, corresponding to the Rh:Mn molar ratios of 1:100, 2.5:100 and 5:100). In the preliminary experiment shown in Figure 1a, M_1_/MnO_x_ (M_1_= Ni, Pt, Pd, Au and Ag) were synthesised following the same procedure, except that the molar ratio of M_1_:Mn was kept at 5:100 in the precursor.

**Synthesis of MnO_x_.** The MnO_x_ catalysts were prepared using the same method mentioned in the preparation of Rh/MnO_x_. Typically, 0.5 mmol of Mn(NO_3_)_2_·4H_2_O (97%, Sigma-Aldrich) and 0.5 mmol citric acid (99.5%, Fisher scientific) were mixed with tiny amount of DI water (~1 mL) to produce a viscous gel under vigorous stirring and heating at 80 °C in an alumina crucible. The as-prepared gel was calcined at 450 °C for 3 h with the heating rate of 5 °C min^–1^. The sample was collected by grinding and denoted as MnO_x_.

**Synthesis of Rh_2.5_/MnO-com**. The Rh_2.5_/MnO-com catalysts were prepared using a wet impregnation method. Typically, a specified amount of RhCl_3_·xH_2_O (38-40% Rh, Sigma-Aldrich) was dissolved in DI water and subsequently added to 100 mg MnO (99%, Sigma-Aldrich) under vigorous stirring in an alumina crucible to achieve a 2.68 wt.% metal loading on MnO. After drying at 60 °C, the prepared mixture was calcined at 450 °C for 3 h with the heating rate of 5 °C min^–1^ in a muffle furnace. The samples synthesised by this impregnation method were denoted as Rh_2.5_/MnO-com.

**Synthesis of Rh_2.5_/Mn_2_O_3_-com**. The Rh_2.5_/Mn_2_O_3_-com catalysts were prepared using the same method mentioned in the preparation of Rh_2.68_/MnO. Typically, a specified amount of RhCl_3_·xH_2_O (38-40% Rh, Sigma-Aldrich) was dissolved in DI water and subsequently added to 100 mg Mn_2_O_3_ (99%, Sigma-Aldrich) under vigorous stirring in an alumina crucible to achieve a 2.68 wt.% metal loading on Mn_2_O_3_. After drying at 60 °C, the prepared mixture was calcined at 450 °C for 3 h with the heating rate of 5 °C min^–1^ in a muffle furnace. The samples synthesised by this impregnation method were denoted as Rh_2.5_/Mn_2_O_3_-com.

**Characterisation.** Powder XRD data were recorded using a Stoe STADI-P diffractometer equipped with a Mo Kα source (wavelength 0.70930 Å, scanned from 2° to 40°, step size 0.5° with 5.0 s per step). Elemental composition was measured using an Agilent 5110 ICP-OES. Diffuse reflectance UV-vis-NIR absorption spectra were collected in the range of 200-2500 nm on a UH5700 spectrophotometer, using BaSO_4_ as the non-absorbing reference. XPS analysis was performed using two spectrometers: a Shimadzu Kratos AXIS SUPRA+ and a Thermo Scientific K-Alpha XPS. The obtained data were analysed with CasaXPS software using the C 1*s* peak at 284.8 eV for calibration. Raman spectra were collected using a Bruker Senterra II Raman spectrometer with a 532 nm laser operated at 0.25 mW. STEM-HAADF images were performed using two instruments: a Thermo Fisher Titan Themis G2 300 operated at 300 kV with an EDX module (used in Figure 3 b-f and Figure S19 a-b), and a Thermo Fisher Scientific Spectra 300 S/TEM operated at 200 kV with a camera length of 115 mm, equipped with a ChemiSTEM EDS detector (used in Figure S23 a-e). TEM images (used in Figure S36 a-b) were performed using a JOEL JEM-2100 electron microscope. EPR measurements were conducted on Bruker EMXplus-6/1. The XAFS (Rh K-edge) were collected at B18 beamline in Diamond Light Source (UK). The storage rings were operated at 3.0 GeV with a stable current of 300 mA. The radiation was monochromatized using a Si (311) double-crystal monochromator. The data collection was carried out in fluorescence mode using a 36-element Ge detector with xspress2 electronics. All spectra were collected under QEXAFS mode in ambient conditions.

**Light-driven dry reforming of methane (DRM) reaction.** The light-driven DRM experiments were conducted using a flow reactor. Typically, the glass fibre membrane was first transferred to the middle of the reactor. A stainless steel round sheet with a central hole (10 mm in diameter) was placed on the glass fibre membrane and secured using a stainless steel ring. The catalyst was then evenly distributed within the central area of the round sheet. Finally, a rubber O-ring and a stainless steel cap were used to seal the reactor. The catalyst was irradiated by a high-powered 300 W Xe lamp (Beijing Perfect Light, PLS-SXE300E) with a concentrator (Thorlabs, N-BK7 Plano-Convex Lens) from the quartz window of the cap. The light spot was controlled to a diameter of 10 mm, ensuring that only the catalyst surface was irradiated. The temperature of the catalyst bed could be monitored and controlled by the thermocouple inserted from the bottom. Four gases, methane (BOC, 99.995%), carbon dioxide (BOC, 99.995%), nitrogen (BOC, 99.998%) and argon (BOC, 99.999%), were supplied to the reactor at different ratios controlled by four mass flow controllers (Bronkhorst). The reaction system was purged by the gas mixture with the setting ratio for at least 120 min to reach equilibrium before photocatalytic activity evaluation. The reactor was connected to gas chromatographs-mass spectrometry (GC-MS: Agilent 8890-5977) for product analysis. In the GC-MS, the thermal conductivity detector (TCD) with a CarbonPlot column and a 5A molecular sieve were used for the analysis of H_2_ and N_2_. In addition, an HT-PLOT Q column connected to the flame ionization detector (FID) was used to detect products, such as CH_4_, CO_2_, CO, C_2_H_4_, C_2_H_6_ and so on. For isotope labelling experiments, isotopically labelled products were analysed using the MS detector.

**In-situ diffuse reflectance infrared Fourier transform spectroscopy (DRIFTS) experiment** **(switching gas mode).** In-situ DRIFTS experiments were performed using a Shimadzu FTIR spectrometer (IRAffinity-1S) with a detector equipped with a Harrick cell and a Praying Mantis accessory. The reaction cell had two ZnSe windows designed for IR transmission. For each test, 10 mg of catalyst was placed in the sample holder. The sample was flattened using a metal spatula to ensure a uniform surface for measurement. Before measurement, the fresh catalyst was purged with a CH_4_/CO_2_/Ar mixture (CH_4_/CO_2_/Ar=4/4/22, GHSV=180,000 mL h^-1^ g_cat_^-1^) at room temperature for 30 min to remove impurities. The temperature was then increased to 500 °C under the mixed gas flow and maintained for 60 min to complete structural transformation. The system was then purged with Ar (GHSV=600,000 mL h^-1^ g_cat_^-1^) for an additional 30 min to remove any residual reactant gases and ensure a clean surface before subsequent measurements. After cooling to room temperature, the system was exposed to a CH_4_ flow (GHSV=180,000 mL h^-1^ g_cat_^-1^) for 30 min. The valves at both ends of the Harrick cell were closed to switch the system to batch mode. The catalyst was then heated from room temperature to 500 °C. DRIFTS spectra were recorded at 400 °C and 500 °C. After reaching 500 °C, DRIFTS spectra were collected at 1, 10, 20, 30, and 40 min. After the first CH_4_ cycle measurement, the system was purged with Ar (GHSV=600,000 mL h^-1^ g_cat_^-1^) for 30 min under 500 °C. A spectrum was collected under Ar to confirm the removal of reactant and product species. After cooling to room temperature, the catalyst was exposed to a CO_2_ flow (GHSV=180,000 mL h^-1^ g_cat_^-1^) for 30 min. After switching to batch mode, the catalyst was heated from room temperature to 500 °C. DRIFTS spectra were recorded at 400 °C and 500 °C. After reaching 500 °C, the DRIFTS spectra of first CO_2_ cycle were collected at 1, 10, 20, 30, and 40 min. After the first CO_2_ cycle measurement, the second CH_4_ cycle measurement was then repeated following the same procedures as mentioned above.

**In-situ DRIFTS experiment for DRM.** In-situ DRIFTS experiments for DRM were performed using in the same equipment mentioned in in-situ DRIFTS experiment (switching gas mode). For each test, 10 mg of catalyst was placed in the sample holder. The sample surface was flattened using a metal spatula to ensure a uniform surface for measurement. Before measurement, the fresh catalyst was purged with a CH_4_/CO_2_/Ar mixture (CH_4_/CO_2_/Ar=4/4/22, GHSV=180,000 mL h^-1^ g_cat_^-1^) at room temperature for 30 min to remove impurities. The temperature was then increased to 500 °C under the mixed gas flow and maintained for 60 min. The system was then purged with Ar (GHSV=600,000 mL h^-1^ g_cat_^-1^) for an additional 30 min to remove any residual reactant gases and ensure a clean surface before subsequent measurements. After cooling to room temperature, a spectrum was collected as the background spectra. Subsequently, the catalyst was exposed to a CH_4_/CO_2_/Ar mixture (GHSV=180,000 mL h^-1^ g_cat_^-1^). DRIFTS spectra were collected at 1, 2, 5, 10, 20 and 30 min in room temperature until no further changes in CH_4_ and CO_2_ adsorption signals were observed. After adsorption equilibrium was reached, the temperature was then increased from room temperature to 500 °C. DRIFTS spectra were recorded at 400 °C and 500 °C. After reaching 500 °C, DRIFTS spectra were collected at 1, 5, 10, 20, and 30 min.

**Surface temperature monitoring experiment.** The surface temperature measurements were carried out using the same reactor as that employed for the light-driven DRM reaction. A total of 10 mg of catalyst was loaded into the reactor, and a CH_4_/CO_2_/N_2_/Ar gas mixture (CH_4_/CO_2_/N_2_/Ar=4/4/2/22, GHSV=192,000 mL h^-1^ g_cat_^-1^) was introduced for 30 min to remove residual impurities in the system. After the light source was turned on, the system was allowed to stabilise under continuous illumination (9.8 W cm^-2^). The catalyst was illuminated for an additional 30 min to ensure steady thermal conditions. During this period, the catalyst was allowed to undergo structural transformation and reach a steady state under the reaction environment before measurement. The surface temperature distribution of the catalyst was recorded using a thermal imaging camera (RS-875, RS PRO).

**Photo-thermo decoupling experiment.** The photo-thermo decoupling experiment was conducted in a Harrick cell. A total of 1 mg of catalyst was placed at the centre of the cell, and a CH_4_/CO_2_/N_2_/Ar mixture (CH_4_/CO_2_/N_2_/Ar=4/4/2/22, GHSV=192,000 mL h^-1^ g_cat_^-1^) was introduced and allowed to flow for 30 min to purge the system. The cell was then heated using an external heating source with a thermocouple positioned at the centre of the catalyst bed to maintain the temperature at 450 °C. Under dark conditions, the temperature was maintained at 450 °C using external heating only. Under illuminated conditions, the catalyst surface was irradiated with an LED light (365 nm, Beijing Perfect Light, PLS-LED 100C) with an irradiance of 0.36 W cm^-2^ while the temperature was maintained at 450 °C by external heating. The reaction was proceeded for 60 min before analysing the products.

**Photo-to-thermo heating verification experiment.** To probe the role of photo-to-thermo heating in the DRM reaction, the experiment was conducted in a Harrick cell. The catalyst was deposited onto a membrane by vacuum filtration to form an ultrathin and uniform catalyst layer with a thickness below 100 μm. The cross-sectional image of the catalyst-loaded membrane was obtained using an optical microscope (OLYMPUS, STM7) to confirm the thickness of the catalyst layer. The catalyst-loaded membrane was then placed at the centre of the cell, and a CH_4_/CO_2_/N_2_/Ar mixture (CH_4_/CO_2_/N_2_/Ar = 4/4/2/22, GHSV = 192,000 mL h^-1^ g_cat_^-1^) was introduced and allowed to flow for 30 min to purge the system. Under concentrated-light conditions, the catalyst-loaded membrane was irradiated with a Xe lamp (Beijing Perfect Light, PLS-SXE300E; irradiance = 9.6 W cm^-2^), and the temperature was monitored using a thermocouple positioned at the centre of the catalyst layer. The temperature was maintained at 480 °C during the test. Under dark conditions, the temperature was maintained at 480 °C using external heating only.

**Temperature-programmed reduction (TPR) experiment.** H_2_-TPR measurements were carried out on a XinHeng chemisorption analyser. Typically, 50 mg of catalyst was loaded into a U-shaped quartz tube reactor. The sample was first purged with a CH_4_/CO_2_ (CH_4_/CO_2_=15/15, GHSV=36,000 mL h^-1^ g_cat_^-1^) gas mixture at room temperature for 60 min. Then, the temperature rapidly increased to 450 °C and held for 60 min under the same flow to complete structural transformation. Afterward, the reactor was purged with He (GHSV=60,000 mL h^-1^ g_cat_^-1^) at 450 °C for another 60 min to remove any remaining gases. After cooling again to 50 °C, a H_2_/Ar mixture (H_2_/Ar=5/45, GHSV=60,000 mL h^-1^ g_cat_^-1^) was introduced for 60 min until a stable baseline was achieved. Finally, the temperature was ramped from 50 °C to 600 °C at a rate of 5 °C min^-1^ under the same H_2_/Ar flow. The reduction process was monitored by TCD.

**Temperature-programmed oxidation (TPO) experiment.** TPO measurements were performed using an AutoChem HP 2950 chemisorption analyser (Micromeritics, USA) equipped with an MKS Cirrus 2 thermal conductivity detector–mass spectrometry (TCD-MS, MKS Instruments, USA) unit. Typically, 50 mg of the spent catalyst (collected after 30 h of DRM reaction in the flow reactor, reaction conditions: 10 mg catalyst; CH_4_/CO_2_/N_2_/Ar = 4/4/2/22; GHSV = 192,000 mL h^-1^ g_cat_^-1^, light wavelength = 200-2500 nm; irradiance = 12.8 W cm^-2^) was loaded into a U-shaped quartz tube reactor. To remove any physically adsorbed species and residual moisture, the sample was first purged with He (GHSV = 150,000 mL h^-1^ gcat^-1^) at 150 °C for 60 min and then cooled to 50 °C. Subsequently, an O_2_/Ar mixture (O_2_/Ar = 5/45, GHSV = 150,000 mL h^-1^ g_cat_^-1^) was introduced until a stable baseline was achieved. Finally, the temperature was ramped from 50 °C to 800 °C at a heating rate of 10 °C min^-1^ under the same O_2_/Ar flow. Desorbed products were detected by TCD-MS.

**^18^O-labelling experiment.** A certain amount of fresh catalyst was loaded into a home-made flow reactor. The reactor could be switched to batch mode by closing the valves at both ends. Prior to the labeling experiment, the reactor was purged with a CH_4_/C^16^O_2_/Ar mixture (CH_4_/C^16^O_2_/Ar = 4/4/22 mL min^-1^) at room temperature for 120 min to remove surface impurities. Subsequently, the catalyst was irradiated (300 W Xe lamp with concentrator, irradiance = 12.8 W cm^-2^) for 60 min under flow conditions. The system was then purged with Ar (100 mL min^-1^) under irradiation for an additional 60 min to remove any residual reactant gases and ensure a clean catalyst surface. A CH_4_/C^18^O_2_/Ar mixture (CH_4_/C^18^O_2_/Ar = 4/4/22 mL min^-1^) was then introduced into the reactor for 20 min. Afterwards, the reactor was sealed by switching off the valves at the inlet and outlet to create a batch mode for measurement. The catalyst was irradiated for 2 min and the reaction products were sampled by a gas-tight syringe, which were analysed by GC-MS (Agilent 8890-5977). Following this measurement, the CH_4_/C^18^O_2_/Ar mixture (CH_4_/C^18^O_2_/Ar = 4/4/22 mL min^-1^) was introduced into the reactor by switching on the valves under irradiation for 20 min to ensure complete substitution of surface lattice ^16^O by ^18^O on the catalyst. The reactor was then purged with Ar (100 mL min^-1^) under irradiation for 60 min to remove any residual reactant gases and ensure a clean catalyst surface. The reactor was subsequently fed with a CH_4_/C^16^O_2_/Ar mixture (CH_4_/C^16^O_2_/Ar = 4/4/22 mL min^-1^) for 20 min. After switching off the valves to establish batch conditions for measurement, the catalyst was irradiated for 2 min and the reaction products were sampled again by a gas-tight syringe, which were analysed by GC-MS.

**Density-functional theory (DFT) computational details.** DFT calculations were performed using the Vienna Ab initio Simulation Package (VASP, version 6.3.2)^[2,3]^ with the Projector Augmented-Wave (PAW) pseudopotentials^[4,5]^. The Generalized-Gradient Approximation (GGA) functional Perdew-Burke-Ernzerhof (PBE)^[6]^ was used throughout. All calculations were spin-polarized and van der Waals dispersion forces were included using the Grimme DFT-D3 method with Becke-Johnson (BJ) damping^[7]^. For bulk calculations of Rh and MnO, a plane-wave cutoff energy of 560 eV was used, and the Brillouin zone was sampled using a Gamma-centered Monkhorst-Pack (MP)^[8]^ grid of 12×12×12. The electronic energy self-consistent convergence threshold was set to 10^-6^ eV and the force convergence criterion was 10^-5^ eV Å^-1^. For calculations involving the catalytic systems with Rh clusters loaded onto the MnO (110) and Mn (111) surfaces, a plane-wave cutoff energy of 450 eV was used, and the Brillouin zone was sampled using Gamma-centered grid MP grids of 2×3×1 and 2×2×1, respectively. The electronic self-consistent energy convergence threshold was 10^-5^ eV and the force convergence criteria was 0.02 eV Å^-1^. A minimum vacuum spacing of 15 Å was maintained between adjacent slabs for both MnO systems, with the MnO (110) slab containing four layers and the Mn (111) slab containing five layers (ensuring that both surfaces are oxygen-terminated). All layers below the top 2 Å for both slabs were kept fixed to represent the bulk layers.

**DRM performance calculation.**

The H_2_ and CO production rates were calculated based on the following equations (N_2_ as an internal standard):

$$f^{out}=\frac{c_{N_{2}}^{in}\times f^{in}}{c_{N_{2}}^{out}}$$

$$R_{H_{2}}=\frac{c_{H_{2}}^{out} \times f^{out}}{V_{m}}$$

$$R_{CO}=\frac{c_{CO}^{out} \times f^{out}}{V_{m}}$$

where $R_{H_{2}}$ is the H_2_ production rate, $R_{CO}$ is the CO production rate, $f^{out}$ is the flow rate of the gas exiting the reactor, $f^{in}$ is the flow rate of the gas entering the reactor, $c_{N_{2}}^{in}$ is the inlet concentration of N_2_, $c_{N_{2}}^{out}$ is the outlet concentration of N_2_, $c_{H_{2}}^{out}$ is the outlet concentration of H_2_, $c_{CO}^{out}$ is the outlet concentration of CO, $V_{m}$ is the gas molar volume under standard conditions.

The CH_4_ and CO_2_ conversion were calculated based on the following equations (corrected by N_2_ as an internal standard):

$$X_{{CH}_{4}}=\frac{c_{{CH}_{4}}^{in}\times f^{in} - c_{{CH}_{4}}^{out}\times f^{out}}{c_{{CH}_{4}}^{in}\times f^{in}}\times100$$

$$X_{{CO}_{2}}=\frac{c_{{CO}_{2}}^{in}\times f^{in} - c_{{CO}_{2}}^{out}\times f^{out}}{c_{{CO}_{2}}^{in}\times f^{in}}\times100$$

where $X_{{CH}_{4}}$ is the conversion of CH_4_, $X_{{CO}_{2}}$ is the conversion of CO_2_, $c_{{CH}_{4}}^{in}$ is the inlet concentration of CH_4_, $c_{{CH}_{4}}^{out}$ is the outlet concentration of CH_4_, $c_{{CO}_{2}}^{in}$ is the inlet concentration of CO_2_, $c_{{CO}_{2}}^{out}$ is the outlet concentration of CO_2_.

The total carbon balance was considered using the following equation:

$$The total carbon balance=\frac{c_{CO}^{out}\times f^{out}}{c_{{CH}_{4}}^{in}\times f^{in}{\times X_{{CH}_{4}}+ c}_{{CO}_{2}}^{in}\times f^{in}\times X_{{CO}_{2}}}\times100$$

**Determination of light-to-chemical energy efficiency.**

The experiments were conducted under the following reaction conditions: 10 mg catalyst, CH_4_/CO_2_/N_2_ = 160/160/2, GHSV = 1,932,000 mL h^-1^ g_cat_^-1^, light wavelength = 200-2500 nm, irradiance = 14.6 W cm^-2^. Under these reaction conditions, the CH_4_ and CO_2_ conversions were maintained below 10% to minimise kinetic limitations and accurately reflect the intrinsic light-responsive properties of the catalyst.^[9,10]^ A high irradiance was employed to fully exploit the photon-to-phonon conversion potential of the Rh_2.5_/MnO_x_ catalyst.

To enable direct comparison with benchmark photothermo catalysts (e.g., Ni/CeO_2_^[11]^, Ru/LaMnFeO^[12]^), the light-to-chemical efficiency was calculated following the same method^[11–14]^. The calculation accounted for contributions from both DRM reaction and reverse water gas shift (RWGS) reaction (A represents DRM reaction rate: CH_4_ + CO_2_ → 2CO + 2H_2_; B represents RWGS reaction rate: CO_2_ + H_2_ → CO + H_2_O):

$$A =\frac{R_{H_{2}}+R_{CO}}{4}$$

$$B =\frac{R_{CO}-R_{H_{2}}}{2}$$

$$Light-to-chemical efficiency (\%) =\frac{A\times{\Delta H}_{DRM}+B\times{\Delta H}_{RWGS}}{I\times S}\times100$$

where $A$ is the reaction rate of DRM reaction, $B$ is the reaction rate of RWGS reaction, $R_{H_{2}}$ is the H_2_ production rate, $R_{CO}$ is the CO production rate, ${\Delta H}_{DRM}$ is the standard enthalpy change of DRM reaction, ${\Delta H}_{RWGS}$ is the standard enthalpy change of RWGS reaction, $I$ is the irradiance, $S$ is the irradiation area. For the Rh_2.5_/MnO_x_ catalyst in this work, $R_{H_{2}}$was 80 mmol h^-1^, $R_{CO}$ was 108 mmol h^-1^, ${\Delta H}_{DRM}$ was taken as 247 kJ·mol^-1^, ${\Delta H}_{RWGS}$ was taken as 41 kJ·mol^-1^, $I$ was 14.6 W cm^-2^ and S was 0.785 cm^2^. The light-to-chemical efficiency was calculated as 29.5%.

Figure S1. (a) Ni 2*p*, (b) Rh 3*d*, (c) Pt 4*f*, (d) Pd 3*d*, (e) Au 4*f* and (f) Ag 3*d* XPS spectra of M_1_/MnO_x_ (taken at before reaction and after 40 min reaction).

Note S1. The chemical states of these elements are summarised as follows:

1. Ni: In the Ni 2*p* XPS spectra, the Ni/MnO_x_ before and after reaction both exhibit Ni^2+^ and Ni^3+^ species (Figure S1a). The Ni^2+^ species are located at 854.9 eV (Ni 2*p*_3/2_) and 872.2 eV(Ni 2*p*_1/2_).^[15]^ The Ni^3+^ species show Ni 2*p*_3/2_ and Ni 2*p*_1/2_ peaks at 856.8 eV and 874.1 eV, respectively. In addition, the two shake-up satellite peaks are observed, which are characteristic feature of Ni^2+^ species arising from multi-electron transition during XPS process.^[16]^ A broad feature observed at around 850 eV is attributed to the Al Kα_3_ satellite contribution originating from the Al X-ray source of the XPS instrument.^[17]^
2. Rh: In the Rh 3*d* XPS spectra, the fresh Rh_2.5_/MnO_x_ sample mainly exhibits Rh^3+^ species, with the Rh 3*d*_5/2_ and Rh 3*d*_3/2_ peaks at 309.5 eV and 314.2 eV, respectively (Figure S1b)^[18]^. After 40 min DRM reaction, the Rh 3*d* region can be deconvoluted into two distinct Rh states. The first component is assigned to metallic Rh^0^, showing characteristic peaks at 307.4 eV for Rh 3*d*_5/2_ and 312.0 eV for Rh 3*d*_3/2_^[19]^. The second component is attributed to partially oxidised Rh species, with the Rh 3*d*_5/2_ and Rh 3*d*_3/2_ peaks appearing at 308.3 eV and 312.9 eV, respectively^[20]^.
3. Pt: In the Pt 4*f* XPS spectra, the fresh Pt/MnO_x_ sample mainly exhibits two Pt species: metallic Pt^0^ and Pt^4+^ states (Figure S1c). Specifically, the metallic Pt^0^ species are located at 71.3 eV (Pt 4*f*_7/2_) and 74.6 eV (Pt 4*f*_5/2_).^[21]^ The Pt^4+^ species display characteristic Pt 4*f*_7/2_ and Pt 4*f*_5/2_ peaks at 74.6 eV and 78.0 eV, respectively. After 40 min DRM reaction, the spent Pt/MnO_x_ only exhibits metallic Pt^0^ species with the Pt 4*f*_7/2_ and Pt 4*f*_5/2_ peaks at 71.3 eV and 74.6 eV, respectively.
4. Pd: In the Pd 3*d* XPS spectra, the fresh Pd/MnO_x_ sample only exhibits Pd^2+^ states (Figure S1d). The Pd^2+^ species show Pd 3*d*_5/2_ and Pd 3*d*_3/2_ peaks at 337.1 eV and 342.3 eV, respectively.^[22]^ After 40 min DRM reaction, the Pd 3*d* spectrum can be resolved into two distinguishable Pd species. The first contribution is attributed to metallic Pd^0^, displaying typical Pd 3*d*_5/2_ and Pd 3*d*_3/2_ signals at 335.4 eV and 340.6 eV, respectively.^[23]^ The second contribution is Pd^2+^ species, showing Pd 3*d*_5/2_ and Pd 3*d*_3/2_ peaks at 337.1 eV and 342.3 eV, respectively.^[22]^ The slight shifts of metallic Pd^0^ and Pd^2+^ to higher binding energies are likely due to the chemical interaction of Pd species with the support.^[24]^
5. Au: In the Au 4*f* XPS spectra, it is noticeable that the Au/MnO_x_ before and after reaction only exhibit metallic Au^0^ with the Au 4*f*_7/2_ and Au 4*f*_5/2_ peaks at 84.3 eV and 88.0 eV, respectively (Figure S1e).^[25]^ Notably, unlike the other M_1_/MnO_x_ catalysts, the Au species remain metallic state before and after reaction.
6. Ag: In the Ag 3*d* XPS spectra, the fresh Ag/MnO_x_ sample primarily displays Ag^+^ species, with the Ag^+^ 3*d*_5/2_ and Ag^+^ 3*d*_3/2_ peaks located at 367.6 eV and 373.6 eV, respectively (Figure S1f).^[26]^ After 40 min DRM reaction, the Ag 3*d* region is attributed to metallic Ag^0^, exhibiting typical peaks at 368.4 eV for Ag 3*d*_5/2_ and 374.4 eV for Ag 3*d*_3/2_.^[27]^

The markedly different DRM activities observed in Figure 1a can be rationalised by considering both the chemical state of the active component under reaction conditions and its intrinsic ability to activate the first C-H bond of methane. For the Ni/MnO_x_ sample, the relatively low DRM activity can be mainly attributed to the predominance of oxidised Ni species under our reaction conditions. Although Ni-based catalysts are widely used in thermocatalytic DRM, their high activity is generally associated with the formation of metallic Ni^0^ sites, which are efficient for CH_4_ dissociation.^[28]^ In our system, however, the reduction conditions is likely insufficient to fully reduce NiO_x_ to metallic Ni^0^.^[29]^ As a result, Ni may remain mainly in an oxidised state during the DRM reaction. The other M_1_ metal containing samples, including Rh, Pt, Pd, Au and Ag, show metallic states after the DRM reaction. However, in contrast to the superior performance of Rh, these metals generally show lower activity under DRM conditions due to their limited capability for C-H bond activation or CH_4_ adsorption^[30,31]^.

Figure S2. Production rates of H_2_/CO and H_2_/CO ratio of Rh/MnO_x_ synthesised from different mole ratios of precursors (Rh:Mn=0:100, 1:100, 2.5:100 and 5:100). (Reaction conditions: 10 mg catalyst; CH_4_/CO_2_/N_2_ = 15/15/2; GHSV = 192,000 mL h^-1^ g_cat_^-1^; light wavelength = 200-2500 nm; irradiance = 12.8 W cm^-2^)

Table S1. Real percentage of Rh over Rh/MnO_x_ detected by ICP-OES.

| Sample | Rh:Mn | Detected loading (wt%) |
| --- | --- | --- |
| Rh_2.5_/MnO_x_ | 2.5:100 | 2.68 |

Figure S3. Production rates of H_2_/CO and H_2_/CO ratio of Rh_2.5_/MnO_x_ in terms of different reactant components. (Reaction conditions: 10 mg catalyst; GHSV = 192,000 mL h^-1^ g_cat_^-1^; light wavelength = 200-2500 nm; irradiance = 12.8 W cm^-2^)

Figure S4. Conversion of CH_4_ and CO_2_ of Rh_2.5_/MnO_x_ in terms of different reactant components. (Reaction conditions: 10 mg catalyst; GHSV = 192,000 mL h^-1^ g_cat_^-1^; light wavelength = 200-2500 nm; irradiance = 12.8 W cm^-2^)

Figure S5. Production rates of H_2_ and CO and H_2_/CO ratio of Rh_2.5_/MnO_x_ in terms of different Ar flow rates. (Reaction conditions: 10 mg catalyst; flow rates of CH_4_/CO_2_/N_2_ = 4/4/2 mL min^-1^; light wavelength = 200-2500 nm; irradiance = 12.8 W cm^-2^)

Figure S6. Conversion of CH_4_ and CO_2_ of Rh_2.5_/MnO_x_ in terms of different Ar flow rates. (Reaction conditions: 10 mg catalyst; flow rates of CH_4_/CO_2_/N_2_ = 4/4/2 mL min^-1^; light wavelength = 200-2500 nm; irradiance = 12.8 W cm^-2^)

Figure S7. Conversion of CH_4_ and CO_2_ of Rh_2.5_/MnO_x_ in terms of different irradiances. (Reaction conditions: 10 mg catalyst; CH_4_/CO_2_/N_2_/Ar = 4/4/2/22; GHSV = 192,000 mL h^-1^ g_cat_^-1^, light wavelength = 200-2500 nm; irradiance = 2.8; 3.2; 4.8; 12.8 and 14.6 W cm^-2^)

Figure S8. Arrhenius plots for CH_4_ conversion rate of the Rh_2.5_/MnO_x_ catalyst under light irradiation. (Reaction conditions: 10 mg catalyst; CH_4_/CO_2_/N_2_/Ar = 4/4/2/22; GHSV = 192,000 mL h^-1^ g_cat_^-1^, light wavelength = 200-2500 nm; irradiance = 2.8; 3.2; 4.8; 12.8 and 14.6 W cm^-2^, corresponding surface temperature = 229, 256, 311, 545 and 579 °C)

The apparent activation energy of CH_4_ was determined by the Arrhenius formula (k = A*exp(-$E_{a}$/$RT$), where $E_{a}$ is the apparent activation energy (kJ·mol^−1^), $R$ is the universal gas constant (8.314 J·mol^−1^·K^−1^), $T$ is the reaction temperature (K). The $E_{a}$ is obtained by fitting the ${lnr}_{{CH}_{4}}$ and $1/T$. The selected temperature range is consistent with those commonly employed in light-driven DRM, such as ~298-457 °C for CoNiRuRhPd/SrTiO_3_^[32]^ and ~398-452 °C for Ru/LaMnFeO^[12]^.

Figure S9. Production rates of H_2_/CO and H_2_/CO ratio of Rh_2.5_/MnO_x_ in terms of three Rh_2.5_/MnO_x_ samples synthesised at different batches. (Reaction conditions: 10 mg catalyst; CH_4_/CO_2_/N_2_/Ar = 4/4/2/22; GHSV = 192,000 mL h^-1^ g_cat_^-1^, light wavelength = 200-2500 nm; irradiance = 12.8 W cm^-2^)

Figure S10. Conversion of CH_4_ and CO_2_ of Rh_2.5_/MnO_x_ in terms of three Rh_2.5_/MnO_x_ samples synthesised at different batches. (Reaction conditions: 10 mg catalyst; CH_4_/CO_2_/N_2_/Ar = 4/4/2/22; GHSV = 192,000 mL h^-1^ g_cat_^-1^, light wavelength = 200-2500 nm; irradiance = 12.8 W cm^-2^)

Figure S11. Raman spectra of the spent Rh_2.5_/MnO_x_ and MnO_x_ catalyst (taken after 40 min time-on-stream). (Reaction conditions: 10 mg catalyst; CH_4_/CO_2_/N_2_/Ar = 4/4/2/22; GHSV = 192,000 mL h^-1^ g_cat_^-1^, light wavelength = 200-2500 nm; irradiance = 12.8 W cm^-2^)

Figure S12. Raman spectra of the spent Rh_2.5_/MnO_x_ catalyst (taken after long-term experiment in Figure 1c). (Reaction conditions: 10 mg catalyst; CH_4_/CO_2_/N_2_/Ar = 4/4/2/22; GHSV = 192,000 mL h^-1^ g_cat_^-1^, light wavelength = 200-2500 nm; irradiance = 12.8 W cm^-2^)

Figure S13. O_2_-TPO-MS profile of Rh_2.5_/MnO_x_ taken after 30 h time-on-stream.

Figure S14. Carbon balance during the stability test of Rh_2.5_/MnO_x_ under the optimised reaction conditions. (Reaction conditions: 10 mg catalyst; CH_4_/CO_2_/N_2_/Ar = 4/4/2/22; GHSV = 192,000 mL h^-1^ g_cat_^-1^, light wavelength = 200-2500 nm; irradiance = 12.8 W cm^-2^)

Figure S15. Zoomed-in area of Figure 1d. (The symbols in Figure S15 represent the CO production rates measured at the final run of the stability test. 11: Rh/TiO_2_^[33]^; 12: Ni/SiO_2_^[34]^; 13: Ni/Ga_2_O_3_^[35]^; 15: Pt-CeO_2_^[36]^; 17: Ni-Si/ZrO_2_^[37]^; 19: Ni@SiO_2_-ZrO_2_^[38]^. Detailed reaction conditions can be found in Table S2-S3).

Table S2. Presentation of Rh_2.5_/MnO_x_ together with other representative photothermo catalytic DRM systems under irradiance levels of the same order of magnitude, which corresponded to a temperature range of 350 to 580 °C

| Catalyst | Reaction conditions | H_2_/CO production rate (mmol g^-1^ h^−1^) | Stability (h) |
| --- | --- | --- | --- |
| **This work: Rh_2.5_/MnO_x_** | **10 mg catalyst, CH_4_/CO_2_/N_2_/Ar = 4/4/2/22, gas flow rate = 32 mL min^-1^, 200-2500 nm, 12.8 W cm^−2^, 545 °C** | **948/992** | **502** |
| Ni/Ga_2_O_3_^[35]^ | 40 mg catalyst, CH_4_/CO_2_/Ar = 20/20/60, gas flow rate = 10 mL min^-1^, UV-vis light, 3 W cm^−2^, 391 °C (external heating) | 12/12.75 | 8 |
| Rh/TiO_2_^[33]^ | 8.6 mg catalyst, CH_4_/CO_2_/Ar = 0.897/0.905/98.198, gas flow rate = 10 mL min^-1^, UV light, 403 °C (external heating) | 21.5/21.2 | 30 |
| Ni/SiO_2_^[34]^ | catalyst containing 30 mg metallic Ni, CH_4_/CO_2_ = 10/10, gas flow rate = 20 mL min^-1^, 300-800 nm, 1.07 W cm^−2^, 550 °C (external heating) | 37/41.5 | 5 |
| Ni/CeO_2_^[13]^ | 25 mg catalyst, CH_4_/CO_2_/Ar = 20/20/60, gas flow rate = 10 mL min^-1^, 320-800 nm, 2.4 W cm^−2^, 472 °C (external heating) | 66.56/59.72 | 230 |
| Ru/BaTiO_3_^[39]^ | 30 mg catalyst, CH_4_/CO_2_/Ar = 10/10/80, gas flow rate = 30 mL min^-1^, 4 W cm^−2^, 460 °C | 121.2/136.8 | 125 |
| Rh/SrTiO_3_^[40]^ | 30 mg catalyst, CH_4_/CO_2_/Ar = 20/20/60, gas flow rate = 30 mL min^-1^, 4 W cm^−2^, 440 °C | 187.7/237 | 100 |
| CoNiFeZnCr-HEO/Rh^[41]^ | 2 mg catalyst, CH_4_/CO_2_/Ar = 10/10/80, gas flow rate = 10 mL min^-1^, 4 W cm^−2^, 480 °C | 242/246 | 200 |
| CoNiRuRhPd/SrTiO_3_^[32]^ | 5 mg catalyst, CH_4_/CO_2_/Ar = 10/10/80, gas flow rate = 20 mL min^-1^, 200-2500 nm, 4 W cm^−2^, 560 °C | 363.8/373.2 | 150 |
| Ni/CeO_2-x_^[42]^ | 2 mg catalyst, CH_4_/CO_2_ = 10/10, gas flow rate = 20 mL min^-1^, 400-700 nm, 0.79 W cm^−2^, 400 °C (external heating) | 240/435 | 50 |
| Ru/TiO_2_^[43]^ | 5 mg catalyst, CH_4_/CO_2_/Ar = 8/8/84, gas flow rate = 50 mL min^-1^, UV-vis-IR light, 10.5 W cm^−2^, 375 °C | 500/475 | 13.3 |
| NiFe nanoalloy/Al_2_O_3_^[44]^ | 5 mg catalyst, CH_4_/CO_2_/Ar = 25/25/50, gas flow rate = 75 mL min^-1^, UV-vis light, 3.62 W cm^−2^, 350 °C | 331/629 | 20 |
| RuNi single-atom alloy^[45]^ | 5 mg catalyst, CH_4_/CO_2_ = 10/10, gas flow rate = 20 mL min^-1^, full-spectrum, 13.5 W cm^−2^, 350 °C | 538.8/642 | 12 |
| Ru/LaMnFeO^[12]^ | 50 mg catalyst, CH_4_/CO_2_/He = 47/47/6, gas flow rate = 30 mL min^-1^, 5.2 W cm^-2^, 500 °C | 514.68/659.04 | 150 |
| Ni/CeO_2_^[11]^ | 200 mg catalyst, CH_4_/CO_2_ = 1/1, gas flow rate = 10 mL min^-1^, 6.8 W cm^−2^, 580 °C | 114.6/114.6 | 800 |

Table S3. Presentation of Rh_2.5_/MnO_x_ together with other representative thermocatalytic DRM systems operating below 600 °C

| Catalyst | Reaction conditions | H_2_/CO production rate (mmol g^-1^ h^−1^) | Stability (h) |
| --- | --- | --- | --- |
| **This work: Rh_2.5_/MnO_x_** | **10 mg catalyst, CH_4_/CO_2_/N_2_/Ar = 4/4/2/22, gas flow rate = 32 mL min^-1^, 545 °C** | **948/992** | **502** |
| Ni-Si/ZrO_2_^[37]^ | 250 mg catalyst, CH_4_/CO_2_ = 30/30, gas flow rate = 60 mL min^-1^, 400 °C | 0.4/0.64 | 15 |
| Pt-CeO_2_^[36]^ | 200 mg catalyst, CH_4_/CO_2_/N_2_ = 5/5/15, gas flow rate = 25 mL min^-1^, 350 °C | 4.5/22.7 | 10 |
| Ni@SiO_2_-ZrO_2_^[38]^ | 100 mg catalyst, CH_4_/CO_2_/N_2_ = 10/10/5, gas flow rate = 25 mL min^-1^, 550 °C | 23/42 | 15 |
| NiZnC/Al_2_O_3_^[46]^ | 100 mg catalyst, CH_4_/CO_2_ = 15/15, gas flow rate = 30 mL min^-1^, 500 °C | 121/219 | 100 |
| Ni/SiO_2_^[47]^ | 50 mg catalyst, CH_4_/CO_2_/N_2_ = 15/15/20, gas flow rate = 50 mL min^-1^, 500 °C | 156.8/313.6 | 33.3 |
| Ni-Fe/Mg(Al)O^[48]^ | 50 mg catalyst, CH_4_/CO_2_/N_2_ = 12.5/12.5/25, gas flow rate = 50 mL min^-1^, 500 °C | 262/390 | 25 |
| Ni/CeO_2_ nanorod^[49]^ | 20 mg catalyst, CH_4_/CO_2_ = 30/30, gas flow rate = 60 mL min^-1^, 500 °C | 297.5/660.5 | 100 |

Table S4. Presentation of Rh_2.5_/MnO_x_ together with other representative photocatalytic DRM systems under low irradiance

| Catalyst | Reaction conditions | H_2_/CO production rate (mmol g^-1^ h^−1^) | Stability (h) |
| --- | --- | --- | --- |
| **This work: Rh_2.5_/MnO_x_** | **10 mg catalyst, CH_4_/CO_2_/N_2_/Ar = 4/4/2/22, gas flow rate = 32 mL min^-1^, 200-2500 nm, 12.8 W cm^−2^, 545 °C** | **948/992** | **502** |
| Cu-C_3_N_4_ nanosheets/Pd-Boron-doped C_3_N_4_ nanosheets^[14]^ | 20 mg catalyst, CH_4_/CO_2_ = 5/5, gas flow rate = 10 mL min^-1^, 200-800 nm, 0.3 W cm^−2^, 35 °C | 0.6613/0.6758 | 40 |
| Ru/TiO_2_-SiO_2_^[50]^ | 30 mg catalyst, CH_4_/CO_2_/Ar = 20/20/60, gas flow rate = 9 mL min^-1^, UV-vis light, 0.975 W cm^−2^, 160 °C | 1.43/1.47 | 20 |
| Rh#CeO_2_^[51]^ | 5 mg catalyst, CH_4_/CO_2_/Ar = 1/1/98, gas flow rate = 10 mL min^-1^, UV light, 0.056 W cm^−2^, 285 °C | 41.8/41.6 | 100 |
| Rh/SrTiO_3_^[52]^ | 5 mg catalyst, CH_4_/CO_2_/Ar = 1/1/98, gas flow rate = 10 mL min^-1^, 150 °C | 57.6/57.6 | 13.3 |

Figure S16. (a) CO production rate over the Rh_2.5_/MnO_x_ catalyst under intermittent operation. (Light-on reaction conditions: 10 mg catalyst; CH_4_/CO_2_/N_2_/Ar = 4/4/2/22; GHSV = 192,000 mL h^-1^ g_cat_^-1^, light wavelength = 200-2500 nm; irradiance = 12.8 W cm^-2^; Light-off conditions: the light irradiation was switched off and the catalyst bed was exposed to ambient air at room temperature) (b) PXRD spectra of Rh_2.5_/MnO_x_ after 12-hour exposure to ambient air and after resuming the DRM reaction.

Figure S17. Comparison of light-to-chemical efficiency with some representative photothermo catalysts (irradiance: 2.4-13.5 W cm^-2^; temperatures: 350-580 °C). (1: CoNiRuRhPd/SrTiO_3_^[32]^; 2: Ru/LaMnFeO^[12]^; 3: Ni/CeO_2_^[11]^; 4: Ni/CeO_2_^[13]^; 5: Rh/SrTiO_3_^[40]^; 6: RuNi single-atom alloy^[45]^; 7: Ru/BaTiO_3_^[39]^; 8: This work)

Figure S18. ^13^C-labeled isotope-labelling and control experiment. (a) Mass spectra after 10 min of reaction using an equivalent amount of ^13^CH_4_ and ^13^CO_2_ as the feeding gas. (b) Mass spectra after 10 min of reaction using an equivalent amount of ^12^CH_4_ and ^12^CO_2_ as the feeding gas. (c) Mass spectra after 10 min of reaction using an equivalent amount of ^13^CH_4_ and ^12^CO_2_ as the feeding gas. (d) Mass spectra after 10 min of reaction using an equivalent amount of ^12^CH_4_ and ^13^CO_2_ as the feeding gas. (Reaction conditions: 10 mg catalyst; CH_4_/CO_2_/N_2_/Ar = 4/4/2/22; GHSV = 192,000 mL h^-1^ g_cat_^-1^, light wavelength = 200-2500 nm; irradiance = 12.8 W cm^-2^)

Figure S19. (a) HAADF-STEM image of Rh_2.5_/MnO_x_. The yellow circles in a highlight the Rh clusters. (b) Zoomed-in region (blue rectangle) in Figure S19a. Figure S19b contains two selected lines #1 and #2, respectively, and their corresponding intensity profiles along the dashed grey rectangles are shown as Figure S19c and Figure S19d.

Figure S20. Experimental and fitted EXAFS data of (a) Rh foil, (b) Rh_2_O_3_ and (c) Rh_2.5_/MnO_x_ at the R space.

Table S5. Rh K-edge EXAFS fitting parameters of Rh foil, Rh_2_O_3_ and Rh_2.5_/MnO_x_.

| Sample | Type | C.N. | R (Å) | σ^2^ | ΔE | R-factor |
| --- | --- | --- | --- | --- | --- | --- |
| Rh foil | Rh-Rh | 12 | 2.69±0.006 | 0.003±0.0003 | 3.4 | 0.006 |
| Rh_2_O_3_ | Rh-O | 6 | 2.04±0.05 | 0.0012±0.0013 | 0.59 | 0.012 |
|  | Rh-Rh | 4 | 2.71±0.003 | 0.003±0.0013 |  |  |
| Rh_2.5_/MnO_x_ | Rh-O_1_ | 1.96 ± 0.11 | 1.95±0.03 | 0.005±0.003 | -0.7 | 0.005 |
|  | Rh-O_2_ | 2.46 ± 0.13 | 2.09±0.02 |  |  |  |

C.N. represents the coordination number, R is the distance to the neighbouring atom, σ^2^ denotes the Mean Square Relative Displacement (MSRD), and ΔE corresponds to the inner potential correction. The R-factor reflects the overall goodness of the fit.

The XAFS data were analysed using the Demeter software package (including Athena and Artemis)^[53–55]^. Athena software was used for XANES analysis. Artemis software was used to fit the k^3^-weighted in real space with 3 Å^-1^ < k < 12 Å^-1^ and 1.0 Å < R < 2.0 Å (for Rh foil and Rh_2.5_/MnO_x_) or 1.0 Å < R < 3.0 Å (for Rh_2_O_3_). The calculated amplitude reduction factor S_0_^2^ from the EXAFS analysis of the Rh foil was 0.83, which was used as a fixed parameter for EXAFS fitting.

Figure S21. PXRD spectra of Rh_2.5_/MnO_x_ before reaction and taken after 0.5, 5 and 40 min time-on-stream.

Figure S22. PXRD spectra of MnO_x_ before and after 40 min reaction.

Figure S23. (a) STEM-HAADF image of Rh_2.5_/MnO_x_ after long-term stability test. (b) High-resolution HAADF-STEM image showing Rh nanoparticles supported on MnO_x_ support after long-term stability test. (c-e) High-resolution HAADF-STEM and EDX images of Rh_2.5_/MnO_x_ after long-term stability test. (f) Particle size distribution of Rh nanoparticles on MnO_x_ support after long-term stability test (Note: 60 particles were counted. The fitting curve in Figure S23f was generated using a Gauss model).

Figure S24. EPR spectra of Rh_2.5_/MnO_x_ after 40 min reaction.

Figure S25. (a) Surface temperature distribution of line scan in Figure 4c. (b) Surface temperature distribution of line scan in Figure 4d. (Screenshot taken from software)

Figure S26. Cross-sectional image of the Rh_2.5_/MnO_x_ catalyst loaded on the membrane.

Note S2. Previous studies on photothermal catalysis have shown that light absorption within catalyst beds is highly surface confined. In powdered catalyst beds, the effective photon penetration depth is often reported to be below approximately 100 μm, while in strongly absorbing photothermo catalysts it can be even shorter, reaching the sub micrometre-scale.^[56–59]^ This indicates that light induced heat generation predominantly occurs within a shallow surface region rather than throughout the entire catalyst bed. Thus, the catalyst was deposited onto a membrane by vacuum filtration to form an ultrathin and uniform catalyst layer. The thickness of the catalyst layer is strictly controlled below 100 μm to ensure that light could penetrate through the entire catalyst layer, as confirmed by the cross-sectional image of the catalyst-loaded membrane using an optical microscope (Figure S26). As illustrated in Figure S27a, two reaction conditions were examined. The CO production rates obtained under the concentrated light and dark conditions are nearly identical at the same catalyst surface temperature of 480 °C (Figure S27b). The comparable CO production rates observed under these two conditions show that the heat generated by light irradiation within this localised micrometre-scale region is sufficient to thermally drive the DRM reaction, and its catalytic effect is effectively equivalent to that achieved by conventional external heating at the same temperature.

Figure S27. (a) Schematic illustration of the experimental setup designed to probe the role of photo-to-thermo heat in the DRM reaction. (b) CO production rates of Rh_2.5_/MnO_x_ catalyst with/without light irradiation at 480°C. (Concentrated-light condition: 1 mg catalyst, surface temperature = 480 °C, light wavelength = 200-2500 nm, irradiance = 9.6 W cm^-2^; Dark condition: 1 mg catalyst, surface temperature = 480 °C, external heating without light irradiation)

Figure S28. H_2_-TPR profiles of TiO_2_, SiO_2_ and MnO_x_ (after activation).

Figure S29. (a-b) In-situ DRIFTS spectra (switching gas mode) over Rh_2.5_/MnO_x_ in 1st CH_4_ cycle. (c-d) In-situ DRIFTS spectra (switching gas mode) over Rh_2.5_/MnO_x_ in 2nd CH_4_ cycle.

Figure S30. MS spectra of CO produced from the normal experiment conditions over Rh_2.5_/MnO_x_ (Reaction conditions: flow rates of CH_4_/C^16^O_2_/Ar = 4/4/22 mL min^-1^; light wavelength = 200-2500 nm; irradiance = 12.8 W cm^-2^).

Figure S31. MS spectra of CO produced from the ^18^O-labelling experiment over Rh_2.5_/MnO_x_ (a) 1st cycle and (b) 2nd cycle.

Figure S32. Production rates of H_2_/CO and H_2_/CO ratio of Rh_2.5_/MnO_x_, Rh_2.5_/MnO-com and Rh_2.5_/Mn_2_O_3_-com. (Reaction conditions: 10 mg catalyst; CH_4_/CO_2_/N_2_/Ar = 4/4/2/22; GHSV = 192,000 mL h^-1^ g_cat_^-1^, light wavelength = 200-2500 nm; irradiance = 12.8 W cm^-2^)

Figure S33. PXRD spectra of Rh_2.5_/MnO-com after 40 min reaction.

Figure S34. PXRD spectra of Rh_2.5_/Mn_2_O_3_-com after 40 min reaction.

Figure S35. O 1*s* XPS spectra of Rh_2.5_/MnO_x_, Rh_2.5_/MnO-com and Rh_2.5_/Mn_2_O_3_-com (taken after 40 min reaction).

Figure S36. (a) TEM image showing Rh nanoparticles of Rh_2.5_/Mn_2_O_3_-com after 40 min reaction. (b) TEM image of Rh_2.5_/Mn_2_O_3_-com after 40 min reaction. (c) Particle size distribution of Rh nanoparticles (Note: 60 particles were counted. The fitting curve in Figure S36 was generated using a Gauss model).

Note S3. Same weight of Rh was loaded to the commercial MnO and Mn_2_O_3_ via a conventional wet impregnation method and their activity have been tested and compared with Rh_2.5_/MnO_x_ (Figure S32). As shown in Figure S32, the Rh_2.5_/MnO_x_ catalyst exhibits significantly higher H_2_ and CO production rates than both reference catalysts prepared using commercial supports (MnO and Mn_2_O_3_). XRD spectra reveal that commercial MnO maintains its phase (Figure S33). In contrast, commercial Mn_2_O_3_ reduces to MnO after the DRM reaction (Figure S34), which is consistent with the structural evolution observed in the Rh_2.5_/MnO_x_ system. Then, the O 1*s* XPS spectra of three catalysts (Rh_2.5_/MnO_x_, Rh_2.5_/MnO-com and Rh_2.5_/Mn_2_O_3_-com) after reaction were obtained to understand the activity difference (Figure S35). The Rh_2.5_/MnO-com catalyst possesses a stable MnO lattice during the DRM reaction with a low O_V_/O_L_ ratio (0.58). In contrast, the *in situ* transformed Mn_2_O_3_ structure in Rh_2.5_/MnO_x_ and Rh_2.5_/Mn_2_O_3_-com can generate more O_V_ during the DRM reaction, thereby they are expected to introduce more defect energy levels within the band structure, which can broaden light absorption and promote the photon-to-phonon conversion channel. Therefore, the higher activity of Rh_2.5_/MnO_x_ and Rh_2.5_/Mn_2_O_3_-com compared with Rh_2.5_/MnO-com has been observed. To further clarify the activity difference between Rh_2.5_/MnO_x_ and Rh_2.5_/Mn_2_O_3_-com, TEM characterisation was conducted to investigate the morphology of the spent Rh_2.5_/Mn_2_O_3_-com catalyst. As shown in Figure S36, the Rh nanoparticles in the spent Rh_2.5_/Mn_2_O_3_-com catalyst have an average size of 4.4 nm, which is larger than that observed in the spent Rh_2.5_/MnO_x_ catalyst (3.6 nm, Figure 3g). This difference can be attributed to the superiority of the synthesis strategy of Rh_2.5_/MnO_x_ in regulating Rh dispersion. In this method, the initial structure of Rh_2.5_/MnO_x_ play a crucial role in constructing the catalytically active architecture. As evidenced by the HAADF-STEM images in Figure S19 and the XAS results in Figure 2b-f, Rh_2.5_/MnO_x_ possesses a solid solution like structure with a homogeneous distribution of Rh on MnO_x_ support. Compared with the Rh_2.5_/Mn_2_O_3_-com synthesised by conventional wet impregnation method, the solid solution structure of Rh_2.5_/MnO_x_ can facilitate the *in situ* formation of more uniformly dispersed smaller Rh nanoparticles under the DRM reaction atmosphere.^[60]^ These highly dispersed small Rh nanoparticles can provide more accessible active sites for CH_4_ activation, which has been identified in our study as the initial step of the DRM reaction. Therefore, the superior activity of Rh_2.5/_MnO_x_ over Rh_2.5_/MnO-com and Rh_2.5_/Mn_2_O_3_-com can be rationalised by the advantage of the current synthesis strategy, which not only constructs oxygen vacancy related defect energy levels to enhance light absorption and promote photon to phonon conversion, but also enables the formation of highly dispersed Rh nanoparticles than the conventional wet impregnation method.

Figure S37. (a-c) In-situ DRIFTS spectra (switching gas mode) over MnO_x_.

Figure S38. All eight adsorption sites for Rh/MnO (110) system: (1a) and (1c) are cluster sites; (2a-2c) and (3a-3b) are all support sites and (1b) is an interface site.

Table S6. Adsorption energies for CH_4_ sites for the Rh/MnO (110) system.

Rh/MnO (110): CH_4_ Adsorption Strengths of Sites (Decreasing order)

| Site Label | Site Type | E_ads_ (eV) |
| --- | --- | --- |
| 1a | Cluster | -0.543 |
| 1c | Cluster | -0.342 |
| 2a | Support | -0.271 |
| 2c | Support | -0.269 |
| 1b | Interface | -0.266 |
| 3a | Support | -0.259 |
| 3b | Support | -0.222 |
| 2b | Support | -0.161 |

Figure S39. All eight adsorption sites for Rh/MnO (111) system: (1a-1b) are cluster sites, (2a-2b), (3a-3b) are all interface sites and (3c-3d) are support sites.

Table S7. Adsorption energies for CH_4_ sites for the Rh/MnO (111) system.

Rh/MnO (111): CH_4_ Adsorption Strengths of Sites (Decreasing order)

| Site Label | Site Type | E_ads_ (eV) |
| --- | --- | --- |
| 1b | Cluster | -0.502 |
| 2b | Interface | -0.342 |
| 2a | Interface | -0.226 |
| 1a | Cluster | -0.172 |
| 3b | Interface | -0.156 |
| 3d | Support | -0.054 |
| 3c | Support | -0.044 |
| 3a | Interface | -0.009 |

Figure S40. Reaction coordinate diagram showing the relative energies of all intermediate and product geometries simulated for each step of the DRM reaction over Rh/MnO (111) system.

Table S8. DFT-Simulated Products and their relative energies along each reaction coordinate (relative to reaction coordinate I) corresponding to Figure S40.

| Reaction Coordinate | DRM Step | Product | Relative Energy to Step-I (eV) |
| --- | --- | --- | --- |
| I | Clean Catalyst + CH_4_ (g) + CO_2_ (g) | 1 | 0 |
| II | CH_4_* + CO_2_ (g) | 1 | -0.502 |
| III | CH_3_* + H* + CO_2_ (g) | 1  2  3  4 | -0.958  -0.718  -0.512  -0.160 |
| IV | CH_2_* + 2H* + CO_2_ (g) | 1  2  3  4 | -0.768  -0.191  0.231  0.838 |
| V | CH* + 3H* + CO_2_ (g) | 1  2  3  4  5 | -0.804  -0.660  0.208  1.050  1.450 |
| VI | CO* + H_2_* + 2H* + O_V_ + CO_2_ (g) | 1  2  3  4  5 | -0.418  0.222  0.290  0.452  1.552 |
| VII | CO* + 2H* + O_V_ + CO_2_ (g)+ H_2_ (g) | 1 | 0.219 |
| VIII | CO* + 2H* + CO_2_* + H_2_ (g) | 1 | 0.975 |
| IX | 2CO* + 2H* + H_2_ (g) | 1 | 0.579 |
| X | Clean Catalyst + 2CO (g)+ 2H_2_ (g) | 1 | 2.338 |

Figure S41. Cluster-site 1b is the most energetically favourable CH_4_ adsorption site for Rh/MnO (111) system. All atomic distances 3 Å or shorter have been displayed.

Table S9. CH_4_ bond lengths (Å) of Rh/MnO (111) system in Figure S41.

|  | 1st C-H | 2nd C-H | 3rd C-H | 4th C-H |
| --- | --- | --- | --- | --- |
| Molecule | 1.09628 | 1.09606 | 1.09606 | 1.09628 |
| Adsorbed | 1.12649 | 1.09597 | 1.11100 | 1.09743 |

Figure S42. DFT calculations of the effect of additional O_V_ on the Rh/MnO (111) system. (a) Rh adsorption energies for Rh/MnO (111) models with different O_V_ configurations. (b) Positions of the additional O_V_ in structures 3A-C in Figure S42a.

Note S4. The Rh adsorption energies were calculated to evaluate how the presence of these additional O_V_ affects the interaction between the Rh cluster and the MnO support (Figure S42a). The positions of the additional O_V_ in structures 3A-C are shown in Figure S42b. In these models, blue denotes the O_V_ involved in the proposed DRM mechanism, while green denotes the additional O_V_ introduced. The results show that the effect of the additional O_V_ depends strongly on its location. For structures 3A and 3B, where the additional O_V_ is located on the exposed surface and in the subsurface or bulk layer, respectively, only minor changes in the Rh adsorption energy and Rh geometry are observed (Figure S42a). This suggests that an increased O_V_ concentration away from the Rh anchoring site does not significantly affect the Rh/MnO system. In contrast, for structure 3C, where the additional O_V_ is located directly underneath the Rh cluster, the Rh geometry is altered and the Rh adsorption energy becomes more favourable. This result indicates that O_V_ located near the Rh anchoring site can modify the Rh-support interaction and may influence the structural stability of the Rh cluster. The more favourable adsorption energy suggests stronger Rh anchoring on the defective MnO support, which may contribute to improved resistance against Rh migration and sintering under DRM conditions.

**References**

[1]	X. Jie, W. Li, D. Slocombe, Y. Gao, I. Banerjee, S. Gonzalez-Cortes, B. Yao, H. AlMegren, S. Alshihri, J. Dilworth, J. Thomas, T. Xiao, P. Edwards, “Microwave-initiated catalytic deconstruction of plastic waste into hydrogen and high-value carbons” *Nat Catal* **2020**, *3*, 902–912.

[2]	G. Kresse, J. Furthmüller, “Efficiency of ab-initio total energy calculations for metals and semiconductors using a plane-wave basis set” *Computational Materials Science* **1996**, *6*, 15–50.

[3]	G. Kresse, J. Furthmüller, “Efficient iterative schemes for *ab initio* total-energy calculations using a plane-wave basis set” *Phys. Rev. B* **1996**, *54*, 11169–11186.

[4]	G. Kresse, D. Joubert, “From ultrasoft pseudopotentials to the projector augmented-wave method” *Phys. Rev. B* **1999**, *59*, 1758–1775.

[5]	P. E. Blöchl, “Projector augmented-wave method” *Phys. Rev. B* **1994**, *50*, 17953–17979.

[6]	J. P. Perdew, K. Burke, M. Ernzerhof, “Generalized Gradient Approximation Made Simple” *Phys. Rev. Lett.* **1996**, *77*, 3865–3868.

[7]	S. Grimme, S. Ehrlich, L. Goerigk, “Effect of the damping function in dispersion corrected density functional theory” *J Comput Chem* **2011**, *32*, 1456–1465.

[8]	D. J. Chadi, M. L. Cohen, “Special Points in the Brillouin Zone” *Phys. Rev. B* **1973**, *8*, 5747–5753.

[9]	Y. Chen, J. Zhao, X. Zhao, D. Wu, N. Zhang, J. Du, J. Zeng, X. Li, M. Salmeron, J. Liu, B. C. Gates, “Stabilizing supported atom-precise low-nuclearity platinum cluster catalysts by nanoscale confinement” *Nat Chem Eng* **2025**, *2*, 38–49.

[10]	X. Chen, M. Wang, Y. He, M. Peng, J. Diao, D. Xiao, N. Wang, X. Cai, H. Liu, D. Ma, “A highly efficient and regenerable Ir1–Cu1 dual-atom catalyst for low-temperature alkane dehydrogenation” *Nat Catal* **2025**, *8*, 436–447.

[11]	Z. Rao, Z. Huang, K. Zhang, J. Wang, Y. Feng, K. Wang, Y. Chen, Y. Cao, L. Li, A. Jiang, K. Zheng, Y. Zhou, “Concentrated-solar catalytic methane dry reforming with ultrahigh conversion and durability” *Nat Commun* **2025**, *16*, 9605.

[12]	J. Li, J. Zhao, S. Wang, K.-S. Peng, B. Su, K. Liu, S.-F. Hung, M. Huang, G. Zhang, H. Zhang, X. Wang, “Activating Lattice Oxygen in Perovskite Ferrite for Efficient and Stable Photothermal Dry Reforming of Methane” *J. Am. Chem. Soc.* **2025**, *147*, 14705–14714.

[13]	Z. Rao, K. Wang, Y. Cao, Y. Feng, Z. Huang, Y. Chen, S. Wei, L. Liu, Z. Gong, Y. Cui, L. Li, X. Tu, D. Ma, Y. Zhou, “Light-Reinforced Key Intermediate for Anticoking To Boost Highly Durable Methane Dry Reforming over Single Atom Ni Active Sites on CeO_2_” *J. Am. Chem. Soc.* **2023**, *145*, 24625–24635.

[14]	W. Zhou, B. Wang, L. Tang, L. Chen, J. Guo, J. Pan, B. Lei, B. Hu, Z. Bai, M. Tulu, Z. Li, X. Wang, C. Au, S. Yin, “Photocatalytic Dry Reforming of Methane Enhanced by ‘Dual‐Path’ Strategy with Excellent Low‐Temperature Catalytic Performance” *Adv Funct Materials* **2023**, *33*, 2214068.

[15]	S. Han, S. Kim, H. J. Cho, J. Y. Lee, J. Ryu, J. Yoon, “Dynamic polarization control of Ni electrodes for sustainable and scalable water electrolysis under alkaline conditions” *Nat Commun* **2025**, *16*, 4803.

[16]	V. V. Kaichev, A. Yu. Gladky, I. P. Prosvirin, A. A. Saraev, M. Hävecker, A. Knop-Gericke, R. Schlögl, V. I. Bukhtiyarov, “In situ XPS study of self-sustained oscillations in catalytic oxidation of propane over nickel” *Surface Science* **2013**, *609*, 113–118.

[17]	J. Clarysse, J. D. J. Silva, Y. Xing, S. B. X. Y. Zhang, S. R. Docherty, N. Yazdani, M. Yarema, C. Copéret, V. Wood, “Earth-abundant Ni-Zn nanocrystals for efficient alkyne semihydrogenation catalysis” *Nat Commun* **2025**, *16*, 4378.

[18]	N. Yang, J. S. Yoo, J. Schumann, P. Bothra, J. A. Singh, E. Valle, F. Abild-Pedersen, J. K. Nørskov, S. F. Bent, “Rh-MnO Interface Sites Formed by Atomic Layer Deposition Promote Syngas Conversion to Higher Oxygenates” *ACS Catal.* **2017**, *7*, 5746–5757.

[19]	N. Zhang, C. He, Y. Jing, Y. Qian, Y. Qin, H. Lin, M. Obuchi, R. Toyoshima, H. Kondoh, K. Oka, L. Li, A. Anzai, T. Toyao, K. Shimizu, “Steam‐Activated Lattice Oxygen Enhances Interfacial Redox Stability for Low‐Temperature N_2_ O Decomposition over Rh/CeO_2_” *Angew Chem Int Ed* **2025**, e202517403.

[20]	Y. Feng, Y. Zhang, J. Wang, L. Ling, R. Zhang, M. Fan, B. Hou, D. Li, B. Wang, “Promotion of Anatase/Rutile Junction to Direct Conversion of Syngas to Ethanol on the Rh/TiO_2_ Catalysts” *ACS Catal.* **2024**, *14*, 1874–1881.

[21]	R. Wang, G. Li, X. Zong, J. Wang, Y. Xu, C. Jin, M. Wang, P. Ma, R. Zhang, K. Zheng, J. Hu, J. Liao, J. Wang, Y. Tang, Y. Dai, S. Wang, S. Wang, “Electron Withdrawal from Methane by Pt Atoms on Stannic Oxide for Highly Active Low-Temperature Combustion” *Environ. Sci. Technol.* **2025**, *59*, 12121–12131.

[22]	G. Tofighi, X. Yu, H. Lichtenberg, D. E. Doronkin, W. Wang, C. Wöll, Y. Wang, J.-D. Grunwaldt, “Chemical Nature of Microfluidically Synthesized AuPd Nanoalloys Supported on TiO_2_” *ACS Catal.* **2019**, *9*, 5462–5473.

[23]	S. Gao, Y. Liu, L. Wang, Z. Wang, P. Liu, J. Gao, Y. Jiang, “Incorporation of Metals and Enzymes with Porous Imine Molecule Cages for Highly Efficient Semiheterogeneous Chemoenzymatic Catalysis” *ACS Catal.* **2021**, *11*, 5544–5553.

[24]	C. Wang, C. Wen, J. Lauterbach, E. Sasmaz, “Superior oxygen transfer ability of Pd/MnOx-CeO2 for enhanced low temperature CO oxidation activity” *Applied Catalysis B: Environmental* **2017**, *206*, 1–8.

[25]	J. Zheng, Y. Lyu, M. Qiao, J. P. Veder, R. D. Marco, J. Bradley, R. Wang, Y. Li, A. Huang, S. P. Jiang, S. Wang, “Tuning the Electron Localization of Gold Enables the Control of Nitrogen‐to‐Ammonia Fixation” *Angew Chem Int Ed* **2019**, *58*, 18604–18609.

[26]	Y. Shiraishi, Y. Shimabukuro, K. Shima, S. Ichikawa, S. Tanaka, T. Hirai, “Sunlight-Driven Generation of Hypochlorous Acid on Plasmonic Au/AgCl Catalysts in Aerated Chloride Solution” *JACS Au* **2023**, *3*, 1403–1412.

[27]	H. Sun, F. Zhang, S. Liu, Y. Liu, B. Zhu, X. Yu, Z. Xie, Z. Zhao, “Insight into the Synergetic Effects of NTP-La _1–_ *_x_* Ag *_x_* MnO_3_ on Soot Oxidation at Low Temperatures” *ACS Catal.* **2024**, *14*, 4927–4943.

[28]	B. Abdullah, N. A. Abd Ghani, D.-V. N. Vo, “Recent advances in dry reforming of methane over Ni-based catalysts” *Journal of Cleaner Production* **2017**, *162*, 170–185.

[29]	L. Sandoval-Diaz, D. Cruz, M. Vuijk, G. Ducci, M. Hävecker, W. Jiang, M. Plodinec, A. Hammud, D. Ivanov, T. Götsch, K. Reuter, R. Schlögl, C. Scheurer, A. Knop-Gericke, T. Lunkenbein, “Metastable nickel–oxygen species modulate rate oscillations during dry reforming of methane” *Nat Catal* **2024**, *7*, 161–171.

[30]	X. Wang, Q. Yuan, J. Li, F. Ding, “The transition metal surface dependent methane decomposition in graphene chemical vapor deposition growth” *Nanoscale* **2017**, *9*, 11584–11589.

[31]	V. Fung, F. (Feng) Tao, D. Jiang, “Low-temperature activation of methane on doped single atoms: descriptor and prediction” *Phys. Chem. Chem. Phys.* **2018**, *20*, 22909–22914.

[32]	H. Xiong, Y. Dong, C. Hu, Y. Chen, H. Liu, R. Long, T. Kong, Y. Xiong, “Highly Efficient and Selective Light-Driven Dry Reforming of Methane by a Carbon Exchange Mechanism” *J. Am. Chem. Soc.* **2024**, *146*, 9465–9475.

[33]	M. Kushida, A. Yamaguchi, M. Miyauchi, “Photocatalytic dry reforming of methane by rhodium supported monoclinic TiO2-B nanobelts” *Journal of Energy Chemistry* **2022**, *71*, 562–571.

[34]	H. Liu, X. Meng, T. D. Dao, L. Liu, P. Li, G. Zhao, T. Nagao, L. Yang, J. Ye, “Light assisted CO_2_ reduction with methane over SiO_2_ encapsulated Ni nanocatalysts for boosted activity and stability” *J. Mater. Chem. A* **2017**, *5*, 10567–10573.

[35]	Z. Rao, Y. Cao, Z. Huang, Z. Yin, W. Wan, M. Ma, Y. Wu, J. Wang, G. Yang, Y. Cui, Z. Gong, Y. Zhou, “Insights into the Nonthermal Effects of Light in Dry Reforming of Methane to Enhance the H_2_ /CO Ratio Near Unity over Ni/Ga_2_ O_3_” *ACS Catal.* **2021**, *11*, 4730–4738.

[36]	D. Shen, Z. Li, J. Shan, G. Yu, X. Wang, Y. Zhang, C. Liu, S. Lyu, J. Li, L. Li, “Synergistic Pt-CeO2 interface boosting low temperature dry reforming of methane” *Applied Catalysis B: Environmental* **2022**, *318*, 121809.

[37]	Y. Wang, L. Yao, Y. Wang, S. Wang, Q. Zhao, D. Mao, C. Hu, “Low-Temperature Catalytic CO_2_ Dry Reforming of Methane on Ni-Si/ZrO_2_ Catalyst” *ACS Catal.* **2018**, *8*, 6495–6506.

[38]	M. Meiliefiana, T. Nakayashiki, E. Yamamoto, K. Hayashi, M. Ohtani, K. Kobiro, “One-Step Solvothermal Synthesis of Ni Nanoparticle Catalysts Embedded in ZrO2 Porous Spheres to Suppress Carbon Deposition in Low-Temperature Dry Reforming of Methane” *Nanoscale Res Lett* **2022**, *17*, 47.

[39]	D. Duan, C. Fu, X. Ji, Z. Dai, A. Chen, C. Hu, M. Ge, H. Xiong, M. Zhou, R. Long, Y. Xiong, “Photoinduced Lattice Oxygen Spillover on Ru/BaTiO_3_ for Efficient and Stable Photothermal Dry Reforming of Methane” *J. Am. Chem. Soc.* **2025**, jacs.5c17614.

[40]	H. Xiong, Z. Dai, C. Fu, X. Ji, Y. Dong, M. Ge, R. Long, Y. Bi, Y. Xiong, “Constructing Dynamic Rh^δ+^ –O_v_ –Ti Interfacial Sites for Highly Efficient and Stable Photothermal Catalytic Methane Dry Reforming” *J. Am. Chem. Soc.* **2025**, *147*, 38204–38214.

[41]	C. Guo, Y. Cui, W. Zhang, X. Du, X. Peng, Y. Yu, J. Li, Y. Wu, Y. Huang, T. Kong, Y. Xiong, “Light‐Driven Metal Exsolution‐Redissolution of High‐Entropy Oxide Enabling High‐Performance Dry Reforming of Methane” *Advanced Materials* **2025**, 2500928.

[42]	K. Lorber, J. Zavašnik, J. Sancho-Parramon, M. Bubaš, M. Mazaj, P. Djinović, “On the mechanism of visible-light accelerated methane dry reforming reaction over Ni/CeO2−x catalysts” *Applied Catalysis B: Environmental* **2022**, *301*, 120745.

[43]	Q. Li, H. Wang, M. Zhang, G. Li, J. Chen, H. Jia, “Suppressive Strong Metal‐Support Interactions on Ruthenium/TiO_2_ Promote Light‐Driven Photothermal CO_2_ Reduction with Methane” *Angew Chem Int Ed* **2023**, *62*, e202300129.

[44]	J. Zhao, X. Guo, R. Shi, G. I. N. Waterhouse, X. Zhang, Q. Dai, T. Zhang, “NiFe Nanoalloys Derived from Layered Double Hydroxides for Photothermal Synergistic Reforming of CH_4_ with CO_2_” *Adv Funct Materials* **2022**, *32*, 2204056.

[45]	Q. Yin, T. Shen, J. Li, C. Ning, Y. Xue, G. Chen, M. Xu, F. Wang, Y.-F. Song, Y. Zhao, X. Duan, “Solar-driven dry reforming of methane using RuNi single-atom alloy catalyst coupled with thermal decomposition of carbonates” *Chemical Engineering Journal* **2023**, *470*, 144416.

[46]	Q. Wang, W. Wang, M. Cao, S. Li, P. Wang, J. He, R. Li, X. Yan, “Effect of interstitial carbon atoms in core-shell Ni3ZnC0.7/Al2O3 catalyst for high-performance dry reforming of methane” *Applied Catalysis B: Environmental* **2022**, *317*, 121806.

[47]	F. Wang, K. Han, L. Xu, H. Yu, W. Shi, “Ni/SiO_2_ Catalyst Prepared by Strong Electrostatic Adsorption for a Low-Temperature Methane Dry Reforming Reaction” *Ind. Eng. Chem. Res.* **2021**, *60*, 3324–3333.

[48]	C. Wan, K. Song, J. Pan, M. Huang, R. Luo, D. Li, L. Jiang, “Ni–Fe/Mg(Al)O alloy catalyst for carbon dioxide reforming of methane: Influence of reduction temperature and Ni–Fe alloying on coking” *International Journal of Hydrogen Energy* **2020**, *45*, 33574–33585.

[49]	K. Lorber, J. Zavašnik, I. Arčon, M. Huš, J. Teržan, B. Likozar, P. Djinović, “CO_2_ Activation over Nanoshaped CeO_2_ Decorated with Nickel for Low-Temperature Methane Dry Reforming” *ACS Appl. Mater. Interfaces* **2022**, *14*, 31862–31878.

[50]	C. He, S. Wu, Q. Li, M. Li, J. Li, L. Wang, J. Zhang, “Constructing matched active sites for robust photocatalytic dry reforming of methane” *Chem* **2023**, *9*, 3224–3244.

[51]	S. Shoji, A. S. Bin Mohd Najib, M.-W. Yu, T. Yamamoto, S. Yasuhara, A. Yamaguchi, X. Peng, S. Matsumura, S. Ishii, Y. Cho, T. Fujita, S. Ueda, K.-P. Chen, H. Abe, M. Miyauchi, “Charge partitioning by intertwined metal-oxide nano-architectural networks for the photocatalytic dry reforming of methane” *Chem Catalysis* **2022**, *2*, 321–329.

[52]	S. Shoji, X. Peng, A. Yamaguchi, R. Watanabe, C. Fukuhara, Y. Cho, T. Yamamoto, S. Matsumura, M.-W. Yu, S. Ishii, T. Fujita, H. Abe, M. Miyauchi, “Photocatalytic uphill conversion of natural gas beyond the limitation of thermal reaction systems” *Nat Catal* **2020**, *3*, 148–153.

[53]	B. Ravel, M. Newville, “*ATHENA* , *ARTEMIS* , *HEPHAESTUS* : data analysis for X-ray absorption spectroscopy using *IFEFFIT*” *J Synchrotron Rad* **2005**, *12*, 537–541.

[54]	H. Funke, A. C. Scheinost, M. Chukalina, “Wavelet analysis of extended x-ray absorption fine structure data” *Phys. Rev. B* **2005**, *71*, 094110.

[55]	H. Funke, M. Chukalina, A. C. Scheinost, “A new *FEFF* -based wavelet for EXAFS data analysis” *J Synchrotron Rad* **2007**, *14*, 426–432.

[56]	X. Li, X. Zhang, H. O. Everitt, J. Liu, “Light-Induced Thermal Gradients in Ruthenium Catalysts Significantly Enhance Ammonia Production” *Nano Lett.* **2019**, *19*, 1706–1711.

[57]	L. Zhou, D. F. Swearer, C. Zhang, H. Robatjazi, H. Zhao, L. Henderson, L. Dong, P. Christopher, E. A. Carter, P. Nordlander, N. J. Halas, “Quantifying hot carrier and thermal contributions in plasmonic photocatalysis” *Science* **2018**, *362*, 69–72.

[58]	X. Zhang, X. Li, M. E. Reish, D. Zhang, N. Q. Su, Y. Gutiérrez, F. Moreno, W. Yang, H. O. Everitt, J. Liu, “Plasmon-Enhanced Catalysis: Distinguishing Thermal and Nonthermal Effects” *Nano Lett.* **2018**, *18*, 1714–1723.

[59]	S. Fang, Y. H. Hu, “Thermo-photo catalysis: a whole greater than the sum of its parts” *Chem. Soc. Rev.* **2022**, *51*, 3609–3647.

[60]	S. M. Sajjadi, M. Haghighi, “Impregnation vs. sol-gel and sol-gel-plasma dispersion of nickel nanoparticles over Al2O3 employed in combined dry reforming and partial oxidation of greenhouse gases to syngas” *International Journal of Hydrogen Energy* **2018**, *43*, 15014–15029.
